# Integrating the behavior of biological soft tissue into musculoskeletal simulation for the design of wearable assistive devices

**DOI:** 10.3389/fnhum.2026.1781987

**Published:** 2026-03-27

**Authors:** David Scherb, Robert Weidner, Sandro Wartzack, Jörg Miehling

**Affiliations:** 1 Engineering Design, Friedrich-Alexander-Universität Erlangen Nürnberg, Erlangen, Germany; 2 Chair for Automated and Autonomous Systems, Technische Universität Bergakademie Freiberg, Freiberg, Germany; 3 Laboratory of Manufacturing Technology, Helmut Schmidt University, Hamburg, Germany

**Keywords:** biological soft tissue, interaction modeling, musculoskeletal simulation, OpenSim, support systems, wearable assistive devices

## Abstract

Wearable assistive devices such as ankle-foot orthoses (AFOs) or exoskeletons are commonly evaluated using musculoskeletal simulations, yet the interface between device and body is typically simplified. In reality, this interface consists of biological soft tissue. Soft tissue deformation influences relative movement, force transmission, and the biomechanical effectiveness of assistive devices. However, this aspect is often neglected in simulation-based design. This study therefore investigates the influence of biological soft tissue on human-device interaction within musculoskeletal modeling. A musculoskeletal simulation model was implemented and extended by integrating an AFO. Biological soft tissue behavior at the device-body interface was represented by viscoelastic spring-damper elements. Simulations were performed using gait data from multiple participants across different walking speeds and AFO support profiles representing varying severities of foot drop. In addition, systematic axis misalignments between the orthosis and the ankle joint were introduced. The simulations revealed that biological soft tissue substantially contributes to relative motion between the AFO and the body segment, with displacements reaching up to 20 mm, particularly near the end of the stance phase during gait. The magnitude of relative movement increased with greater muscle weakness and higher required support forces. In contrast, gait speed and axis misalignment produced comparatively smaller effects. Soft tissue deformation also influenced muscle activation patterns, especially in severely weakened conditions, where deviations of up to 13% were observed in the plantarflexor muscles. The findings demonstrate that incorporating biological soft tissue behavior significantly affects predicted human-orthosis interaction and muscle activation in musculoskeletal simulations. Accounting for these deformations potentially improves the realism of simulation-based analyses and supports more accurate evaluation of assistive device performance. Consequently, explicit modeling of biological soft tissue should be considered essential for the development and optimization of effective and comfortable wearable assistive technologies.

## 1 Introduction

Wearable assistive devices (WADs) are specific products that support or assist the human body by force transmission (Argubi-Wollesen and Weidner, 2018; Miehling et al., 2018; Nguyen and Zhang, 2020). Typical examples are exoskeletons in industrial applications to prevent damage for the human body due to occupational tasks (Looze et al., 2016; Bogue, 2018) aiming for support of body regions like shoulder exoskeletons (Otten et al., 2018; Bock et al., 2022) or back exoskeletons (Toxiri et al., 2019; Kermavnar et al., 2021). Furthermore, there are devices within a medical background that allow the enabling of lost motion patterns and behavior or reduce pain caused by diseases, like orthoses, e.g., ankle-foot orthoses (AFOs) for correcting motion patterns during gait (Blaya and Herr, 2004; Ferris et al., 2005; Alam et al., 2014). Lastly, there are therapeutical devices to help regain normal muscle functionalities, such as upper limb rehabilitation devices for elbow and wrist training purposes (Song and Guo, 2012).

The design of WADs is aimed to be user-centered to provide optimal support for the human and an ergonomic human-WAD-interaction. For this purpose, the manufacturing of product prototypes is quickly realized during the design phase and user studies are performed with these. By doing so, user individual feedback and ergonomic classifications can be gathered (Meyer et al., 2019; Laffranchi et al., 2021). This information can then further be used to optimize the WAD regarding the user requirements. In addition, also quantitative data can be gathered and used for WAD design evaluation and optimization. Information like oxygen consumption or heart rate can be measured to identify the level of exhaustion for the human (Danielsson and Sunnerhagen, 2004; Asselin et al., 2015; Schubert and Weidner, 2025). Furthermore, biomechanical parameters can be utilized to evaluate the effect on the human body. One exemplary parameter is the muscle activity representing a decreased load and stress on the body (Bosch et al., 2016; Alemi et al., 2019). Other examples are parameters affecting the motion behavior, like occurring joint moments, or motion-defining parameters, e.g., gait speed, stride length, cadence etc., can be used to evaluate the efficiency of WADs (Lewis and Ferris, 2011; Böhm et al., 2025).

However, the general direction for the (user-centered) design process of WAD is the shift more toward the virtual world, in terms of the virtual product development (Lashin and Stark, 2021). The vision is to represent the complete product development process into the virtual world. This shift upstream has the greatest impact on product costs and times and also achieves higher product quality (Eigner et al., 2014). Thus, the design process of WADs is also assisted by virtual design tools, like computer-aided design or Finite Element Analysis (FEA; Chu et al., 1995; Perry et al., 2007). A further important factor is also that the effects of WAD on the user can be represented in the virtual world. For human representation, digital human models can be used (Chaffin, 2005). A special sub-group among them are musculoskeletal human models (MHMs). MHMs are virtual representations of the human body that are based on multi-body dynamics (Damsgaard et al., 2006; Seth et al., 2018). The body parts and segments (mainly the bones) are defined as rigid bodies and the muscles act as line actuators. With this setup, they allow biomechanical simulations of executed motions, like analyses of occurring muscle activations and forces or joint reaction forces. For product design, this can be used to develop the design of products or the adjustment of product specifications toward the users' needs from a biomechanical perspective (Wolf et al., 2022; Scherb et al., 2023a).

The use of MHMs for simulations with WADs has emerged in recent years (Scherb et al., 2023c), depicting the biomechanical effects of the interaction between human and WAD design. The possible advantages are the decrease of cost and time due to the reduction of effort and time to manufacture prototypes and perform user studies. Furthermore, biomechanical data that is hard (e.g., surface electromyography) or impossible (e.g., joint reaction forces) to measure can be quantified in an easy and accessible way. Further advantages are that the effects of multiple configurations of the device can be tested easy and quickly, more technical knowledge about the WAD can be gained and no ethical or legal restrictions are existing (Agarwal et al., 2013; Ferrati et al., 2013; Dembia et al., 2017). Thus, the investigation of effects on the biomechanical parameters, like the muscle activation, can be done in a more methodological and detailed way. For example, the influence of different support curves by an AFO for the activation of lower leg muscles during the gait (Scherb et al., 2023b) or the influence of different support characteristics by a shoulder exoskeleton at over-head screwing for the shoulder and arm muscles (Molz et al., 2024) can be investigated. Based on these discovered effects, design implications and adjustments can be derived. This can be implications like an altered dimensioning of structures, material adjustments, different spring stiffnesses or others (Agarwal et al., 2010; Steck et al., 2024b).

However, this process, whereby real design and layout decisions are made based on the previously virtual simulation results of human simulation, is only applied to a limited extent in a few isolated examples (Font-Llagunes et al., 2011; Tröster et al., 2020; Christensen et al., 2021). MHMs are mainly used to evaluate the effects of a WAD prototype biomechanically. After a prototype has been manufactured, it is tested on the user and the biomechanical effectiveness of the WAD is demonstrated through simulation with MHMs (Imamura et al., 2011; Ferrati et al., 2013; Kruif et al., 2017; Michaud et al., 2019). The MHMs therefore serve as an evaluation tool toward the end of product development or for checking the biomechanical impact of the prototype and are not used during the development of the prototype. This shows that the development of WAD still relies heavily on the use of physical user tests (Shorter et al., 2011; Patzkowski et al., 2012; Amandels et al., 2019; Meyer et al., 2019).

The reason for the limited use of MHMs to derive real design decisions could lie in the insufficient representation of human-WAD-interaction in the simulation. For a valid transfer of the findings to the real world, all effects that occur in reality should be reproduced in the simulation as best as possible (Ferrati et al., 2013; Fournier et al., 2018). An important factor that has a major influence on the effective support provided by the WAD is the biological soft tissue at the interface between humans and the WAD (Pons, 2010; Quinlivan et al., 2015; Sánchez-Villamañán et al., 2019). This human-machine interface flexibility can effectively result in the loss of force components intended to provide support (Asbeck et al., 2015; Yandell et al., 2017). The primary effect of the yielding behavior of biological soft tissue is a resulting relative movement between the person (or body part) and the WAD as a result of the force applied by the WAD (Schiele and van der Helm, 2006). This relative movement shifts the effective point of application of the force, resulting in a change in effect due to the support provided by the WAD.

The existing interaction modeling approaches between WAD and MHM in musculoskeletal human simulation examined by Scherb et al. (2023c) reveal that the influence of the biological soft tissue has not yet been integrated. Only kinematic constraints (Schiele and van der Helm, 2006; Zanotto et al., 2015) caused by the misalignment of the WAD and human joint axes (Steck et al., 2024a) are included for the creation of relative movements. Furthermore, the modeling of relative movement reveals a limitation with regard to the permitted degrees of freedom of relative movement at the respective interfaces. Consequently, a maximum of one permitted degree of freedom is usually modeled in musculoskeletal human simulation. However, the investigation of effects of the different identified interaction modeling approaches shows clear implications on the simulation results of human simulation even with limited realization of relative movement [in the investigated example with an AFO on the muscle activation of the plantar flexors (Scherb et al., 2025)]. This depicts that varying insights can be gained from human simulation depending on the interaction modeling approach chosen, especially when considering the relative movement between WAD and MHM. However, the resulting relative movement does not depend exclusively on the kinematic dependence between WAD and body part and is not expressed in just one degree of freedom. Instead, the resulting relative movement is influenced by the kinematic dependence due to the yielding behavior of the biological soft tissue at the interface and is free in all degrees of freedom. By integrating this behavior into human simulation, further real-world effects could be taken into account. This could further improve the virtual design of the WAD and the transfer of the simulation findings to the real product, making it potentially more valid.

Thus, a novel interaction modeling approach should be introduced in this publication that integrates the behavior of the biological soft tissue into the musculoskeletal human simulation for the design of WADs. In particular, the approach should enable the simulation of the yielding behavior of the biological soft tissue at the human-WAD-interaction that should further allow the derivation of design decisions for WADs. The related research question that should be answered in this contribution is:

How can the influence of the biological soft tissue on the force transmission of WADs be integrated in the musculoskeletal human simulation?

## 2 Materials and methods

### 2.1 Representation of biological soft tissue in simulations

In order to investigate the effect of biological soft tissue on the relative movement occurring at the interface between humans and WAD, the soft tissue must be virtually represented in the musculoskeletal simulation. There are two main options for this in the literature: FEA (Nguyen et al., 2020) and spring-damper elements (Omar et al., 2022). Spring-damper elements represent the viscoelastic behavior of material by combining a spring (ideal elastic behavior) and a so-called Newton element (ideal viscous behavior). Spring-damper modeling offers the advantages of simple modeling and significantly reduced computing time. However, this is theoretically accompanied by a loss of accuracy in the simulation of behavior (Murai et al., 2017; Omar et al., 2022). In these studies, the biological soft tissue was constructed from several spring-damper elements representing the individual components of the soft tissue, and the behavior and force effects of these models were examined. This showed good reproduction of data determined in the literature, particularly with regard to compression behavior, creep and relaxation in the linear range. Furthermore, due to the existing use of spring-damper models in muscle models of MHMs (Thelen, 2003; Millard et al., 2013) these models can be easily integrated into musculoskeletal human simulation.

### 2.2 Modeling of biological soft tissue in musculoskeletal simulation of wearable assistive devices

**Figure 1:**
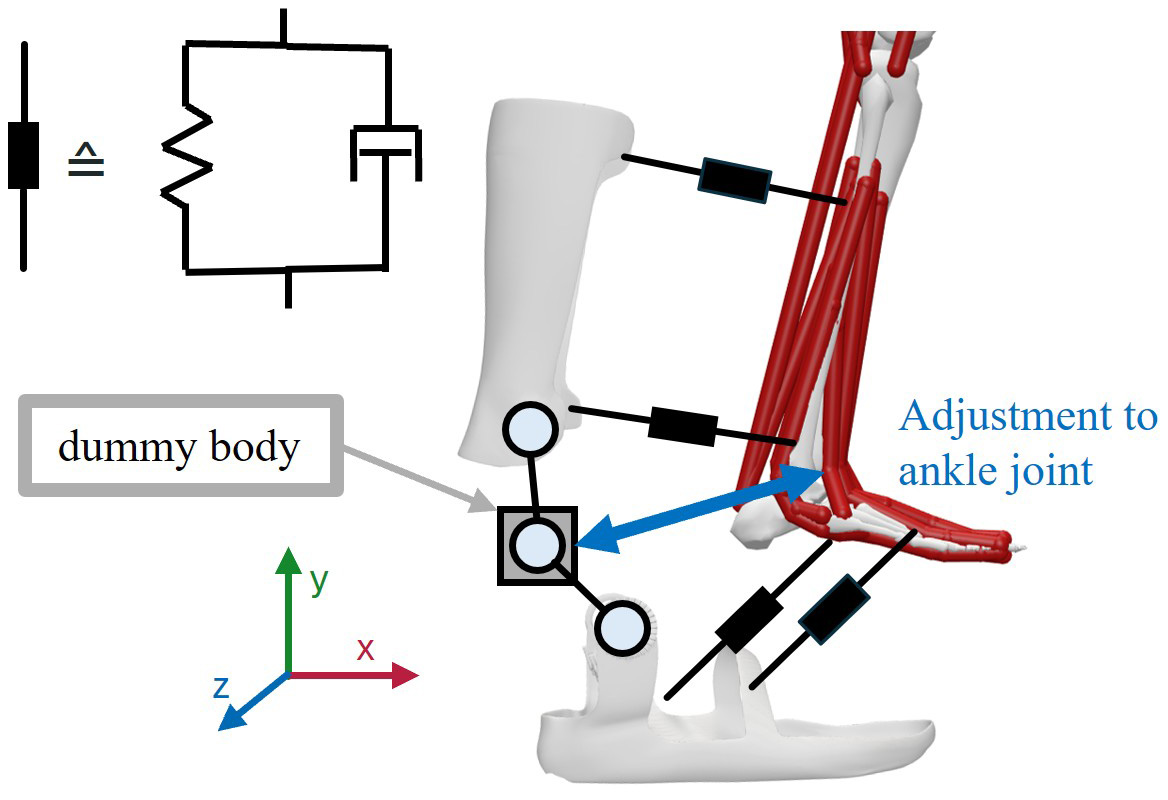
Overview of the modeling concept for the ankle orthosis on the human model using spring-damper elements.

The method for biological soft tissue modeling and simulation to determine the resulting relative movement between MHM and WAD is applied using the example of a passive AFO to support patients with existing foot drop (Stewart, 2008). Particular focus is placed on the effect achieved on force transmission and the resulting effects on the biomechanical simulation results, i.e., on muscle activation. The modeling of biological soft tissue at the interface body areas is also demonstrated here as an example (Figure 1), which can also be adapted to other applications.

In the first step, the AFO model is integrated into the musculoskeletal simulation environment of OpenSim and connected to the MHM with the implementation of biological soft tissue using spring-damper elements. For the initial connection to the MHM, a dummy body is connected to the ankle joint via a CustomJoint, which in the default setting corresponds to the center of rotation of the ankle joint. The dummy body is a virtual, invisible and low-mass (weight: 1e-05 kg) auxiliary body in OpenSim. The dummy body is integrated into the musculoskeletal simulation to allow an easier adjustment of the orthosis axis and ankle joint axis (in detail in Section 2.3.2). The defined CustomJoint allows translations in all three spatial directions, but is locked with regard to rotational degrees of freedom. This prevents rotation of the orthosis as a result of rotation of the dummy body. The foot and calf shells are each connected to the dummy body via a CustomJoint, whose connection point in both shells is defined in the center of rotation of the AFO. The CustomJoints allow rotation around the z-axis in both cases, i.e., they are defined in accordance with the possible rotation in the upper ankle joint. In reality, the orthosis is mainly attached to the patient using straps on the foot (in the area of the instep—as indicated by the bracket in Figure 1) and on the tibia (as indicated by the two protrusions in Figure 1). At this point, it should be noted that a simple connection between the MHM and AFO using CustomJoints at these points is not feasible due to the problems associated with the kinematic chain in the musculoskeletal modeling. Instead, a BushingForce is used in OpenSim to connect the MHM and AFO. A BushingForce is a force actuator in OpenSim that is defined between two bodies and counteracts the deviation of the two bodies with a force. The BushingForce is implemented consisting of three translational and three rotational spring-damper elements. In the example of the AFO, two bushingForces are defined between the body parts and the orthosis shells (black in Figure 1).

The bushing forces, or more generally the spring-damper elements, are defined by stiffness values in the rotational (Nm/rad) and translational (N/m) directions, as well as by damping values in the rotational [Nm/(rad/s)] and translational [N/(m/s)] directions. The same stiffness parameters are used for the two bushing forces between the foot and foot shell and the two bushing forces between the tibia and calf shell. These are shown in Table 1. The parameters are based on existing studies in which methods for determining the stiffness of biological soft tissue during movements in general (Guitteny et al., 2022) as well as leg orthoses (Mouzo et al., 2020) and exoskeletons (Shafiei and Behzadipour, 2020) have already been applied. When selecting the parameters, care was taken to ensure that the values were of a similar magnitude to those in the studies mentioned.

**Table 1:** Translational and rotational stiffness parameters for the different spatial directions for the bushing forces on the calf and foot.

Stiffness						
Tibia	Translational [N/m]			Rotational [Nm/rad]		
	x	y	z	x	y	z
	100	10,000	100	200	500	200
Foot	Translational [N/m]			Rotational [Nm/rad]		
	x	y	z	x	y	z
	10,000	500	500	500	200	200

For modeling purposes, higher stiffness means that greater resistance is encountered. For this reason, the highest value of 10,000 N/m was assumed in the translational direction on the tibia in the y-direction and on the foot in the x-direction, as the straps, bands, Velcro fasteners, etc. fix the ankle brace in place and counteract any displacement with greater force. In comparison, less resistance is applied against displacement in the other two directions. There is significantly more biological soft tissue on the tibia than on the foot. As a result, significantly greater compression of the biological soft tissue can occur on the tibia. Accordingly, the resistance to deformation is also lower. For this reason, the stiffness at the tibia is set at 100 N/m, which is lower than at the foot, where it is set at 500 N/m. The same principle is applied to rotational stiffness. Rotational displacement around the x-axis at the foot and the y-axis at the tibia should be counteracted by the greatest possible resistance from the straps, ligaments, Velcro fasteners, etc., which is why a stiffness value of 500 Nm/rad is used there. In the other directions, the force against rotational displacement is reduced, which is why a stiffness value of 200 Nm/rad is selected for all these directions, both at the calf and at the foot. A damping value of 5 Nm/(rad/s) or N/(m/s) is selected for all bushing forces in all rotational and translational degrees of freedom.

### 2.3 Simulation of biological soft tissue behavior with WAD support

After modeling the biological soft tissue at the interface between MHM and AFO, the next step is to simulate the behavior of the soft tissue during the interaction. The behavior and the occurring effects of the biological soft tissue is directly determined by the application and release of force by the AFO, i.e., by the behavior of the AFO. This behavior is mainly defined by the support curve provided of the AFO. Thus, at first, the support by the AFO has to be specified.

#### 2.3.1 Orthosis support for patient gait

Since the AFO should be used for treating foot drop and its effect on the gait of humans, the gait behavior of participants was recorded in a motion lab. The gait of 15 healthy participants (age: 30 ± 12 years, height: 1.76 ± 0.1 m, weight: 72 ± 11 kg, BMI: 23 ± 1.8 kg/m^2^, 7 females, 8 males) was recorded in a study at three self-selected gait speeds referred to “slow,” “medium,” and “fast.” The detailed description of the study setup, used equipment and data processing is given in Scherb et al. (2023b). In that study, the assistance-as-needed support for optimal treatment by an AFO for varying foot drop severity was determined. For that, from the captured participants different MHMs with varying levels of weakened and paralyzed lower leg muscles, in order to represent foot drop patients, were created. Thus, the dorsiflexor muscles were paralyzed in the MHMs (0% maximum isometric force) and the plantarflexor muscles were adjusted in steps from 25% (PF25), 50% (PF50), 75% (PF75) to 100% (PF100) remaining maximum isometric force. Therefore, from every recorded healthy participant four patient MHMs with varying foot drop severity (PF25 as the weakest to PF100 as the strongest) were created. Afterwards, within the simulative approach, different support curves by the AFO were applied to the MHMs causing different plantarflexor muscle activations. These resulting muscle activations were then compared with the plantarflexor muscle activations of the healthy participants, with the one having the best agreement being classified as the ideal support from an AFO. With this method, for every patient MHM with varying foot drop severity the required support curve from an AFO was identified at each gait speed.

#### 2.3.2 Simulation procedure

The behavior of the AFO must be predicted or determined within the simulation under the effect of the support. For this reason, the computed muscle control simulation (CMC) is used to simulate the behavior of biological soft tissue. The CMC is primarily used to determine muscle activity based on movement and external forces (and can calculate muscle activation from this). However, it is also able to adjust the movement if necessary and determine missing joint angles itself if the boundary conditions are sufficiently defined. This property is used in this biological soft tissue simulation. Consequently, for the simulation, the recorded gait movements of the healthy subjects at the three gait speeds are used for each patient MHM, and the respective determined support curve is applied to the AFO.

**Figure 2:**
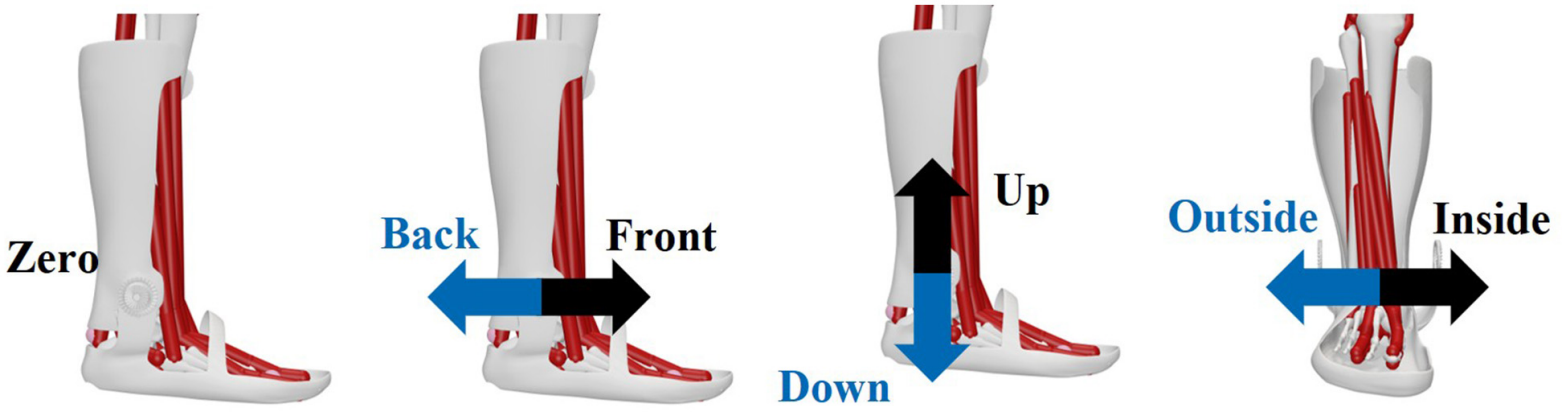
Simulated shifts of the orthosis axis relative to the ankle joint axis, starting from exact alignment (zero) in the following directions: anterior (front), posterior (back), proximal (up), distal (down), medial (inside), and lateral (outside).

The alignment of the orthosis axis relative to the ankle joint axis is a possible factor influencing the relative movement between the MHM and AFO resulting from the influence of biological soft tissue. The misalignment can result in different relative movements and correspondingly different effects on the biomechanical parameters. To investigate this influence, various misalignments are considered in the simulation. In the initial setting, the two axes are modeled as coinciding, which is subsequently referred to as ‘zero'. For the individual misalignments, the displacements of the two axes relative to each other are integrated into the modeling by shifting the dummy body relative to the ankle joint axis. The displacement of the orthosis axis (see Figure 2) is carried out in six directions: proximal (up), distal (down), anterior (front), posterior (back), medial (inside) and lateral (outside), each by 2 cm. A displacement of 2 cm was chosen because, after analyzing various studies, this was identified as the maximum displacement of the axes (Brinckmann et al., 2007; Fatone et al., 2016; Bessler-Etten et al., 2022; Böhm et al., 2025).

**Figure 3:**
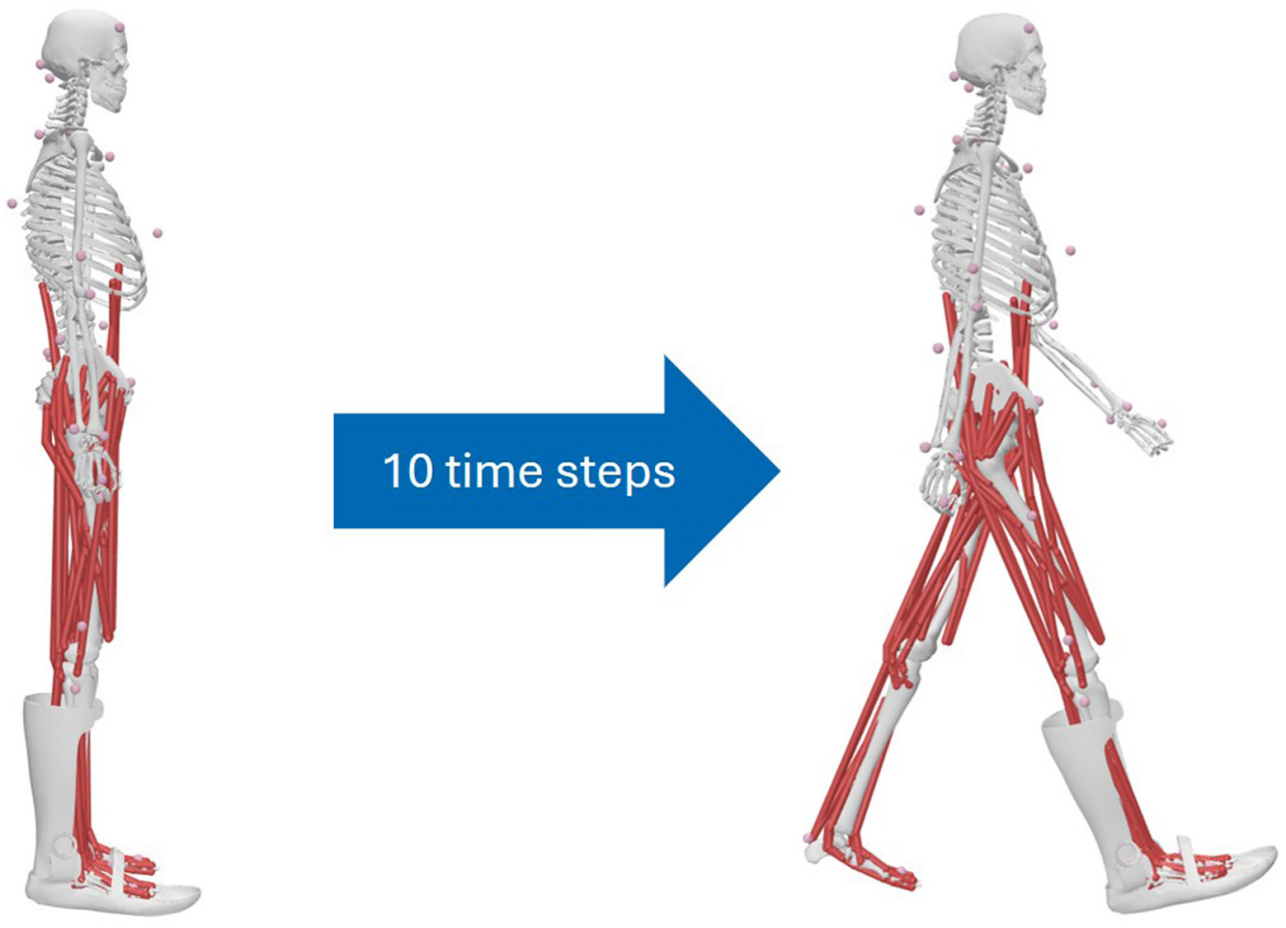
Schematic representation of the initial CMC for generating the initial state of gait movements.

After modeling the various displacements of the AFO for different patient MHMs, the AFO is integrated into the initial state of the MHMs. However, this results in a simulation problem for the CMC. The initial pose of the gait cycle differs so much from the initial state of the MHMs that the CMC cannot automatically identify an initial condition for the AFO position at the start of the gait simulation. Therefore, the initial condition of the AFO must first be determined in the first time step of the gait cycle simulation. To do this, an initial CMC is performed before the actual gait simulation, which is referred to as the ‘initial CMC'. For this initial CMC, a movement is first generated synthetically, with which the MHM moves from the initial pose to the initial pose of the gait movement (see Figure 3). Specifically, in this synthetic movement, the joint angles of the MHM are adjusted linearly from their standard value to the respective initial value of the gait movement within 10 time steps. This initial CMC also simulates the ankle brace (or rather its own degrees of freedom and the degrees of freedom for connection to the MHM) with regard to the initial state of the gait movement. The influence of external forces is still disregarded for the generation of the initial movement. Only virtual force generators on the pelvis support the MHM in the environment, which means that the movement of the lower body can be regarded as being performed in a floating state.

The initial movement is performed for each test participant and for each integrated displacement of the AFO using the basic model (unweakened—obtained from the gait recording in Scherb et al., 2023b). This ensures that the initial state of the gait movement is identical for each weakened state and each displacement. Based on the initial state of the MHMs with orthosis, the CMC of the gait cycle can now be performed. In addition to the ground reaction forces recorded from the gait recording, the specific support required by the ankle AFO is also applied as an external force (from Section 2.3.1). This support torque is applied at the center of rotation of the orthosis and acts accordingly on the MHM via the bushing forces, depending on the resulting relative movement from the set alignment or the deviations of the ankle joint and orthosis axis. As a special setting, the CMC tasks are increased in the CMC of the knee and ankle joint angle of the orthosis side to evaluate the simulated gait motion. The knee and ankle joint angles are therefore weighted with 10. This is to ensure that the adjustment of the gait movement by the forces applied by the ankle orthosis does not lead to any extreme changes in gait behavior. To evaluate the CMC and thus the effects of biological soft tissue influence, the resulting relative movement between MHM and WAD is first analyzed. Finally, the effects on the muscle activations of the plantar flexors are evaluated.

## 3 Results

### 3.1 Relative movement

**Figure 4:**
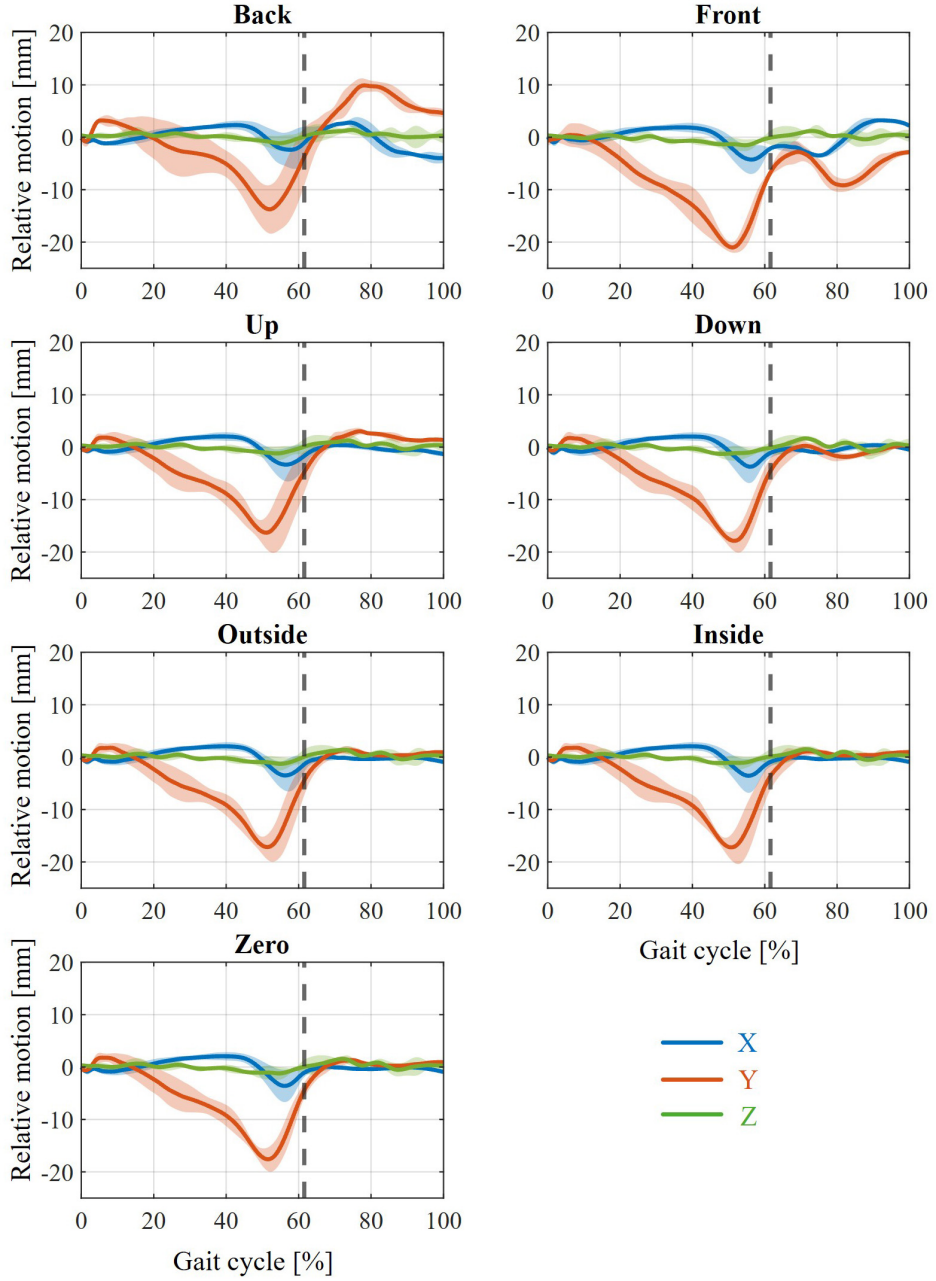
Resulting relative movement between foot and foot shell across all subjects with PF25 for the various shifts between joint and orthosis axis and slow walking speed.

**Figure 5:**
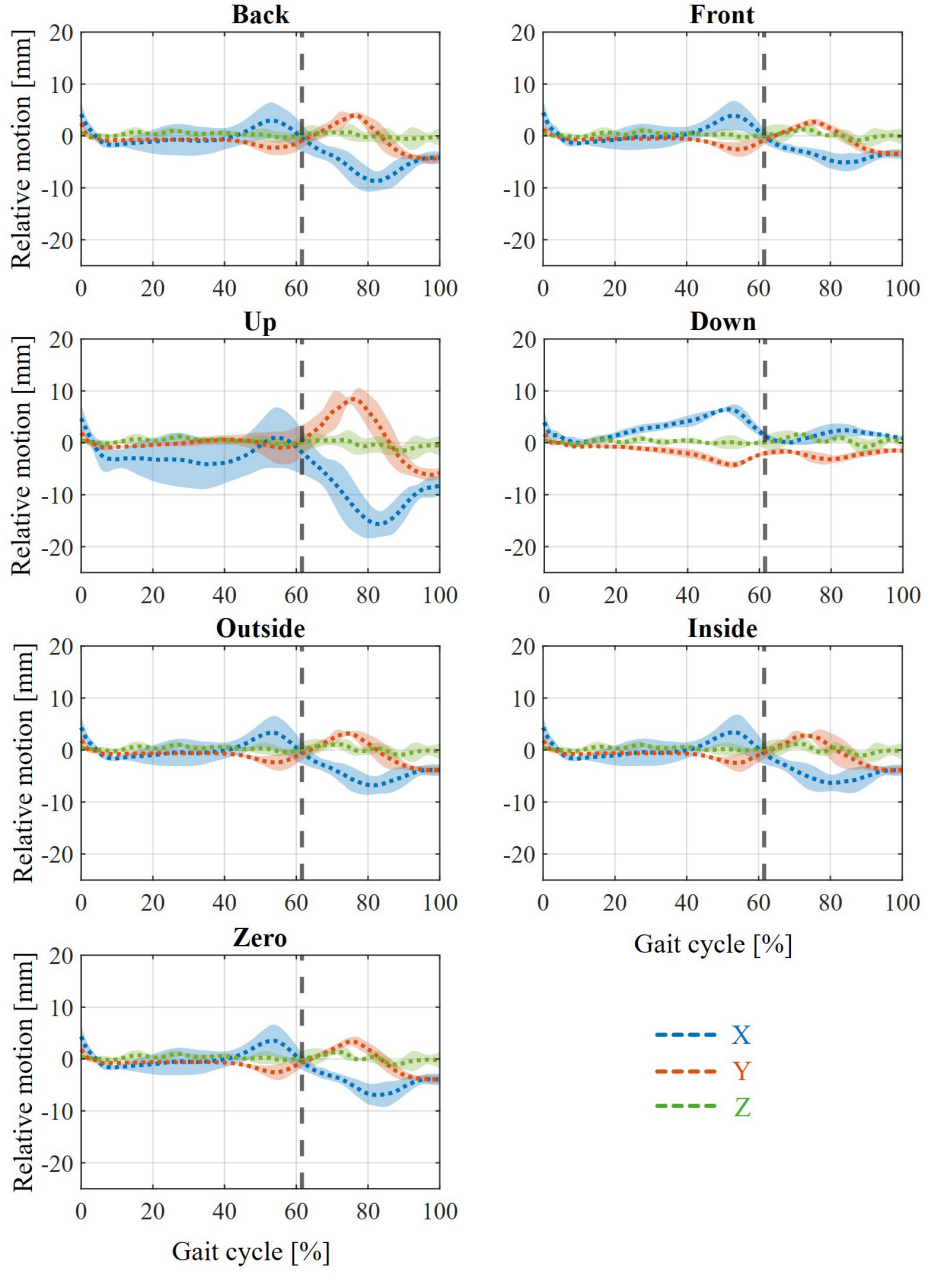
Resulting relative movement between tibia and calf shell across all test subjects with PF25 at various displacements between joint and orthosis axis and slow walking speed.

In the first step, the resulting relative movement resulting from the modeled interaction between the orthosis and the human leg is analyzed. For this purpose, a marker is defined at the origin of the foot shell in the initial state of the model. Another marker is also defined at this position for the toe body of the MHMs, so that both markers coincide in the initial state of the model. A similar procedure is also applied to the calf shell and the tibia body. The relative movement at both positions results from the displacement of the corresponding markers relative to each other. The average relative movement between the foot and the foot shell in state PF25 is examined in the x, y and z directions (the direction definitions are shown in Figure 1) and is illustrated in Figure 4 for the different displacements of the joint and rotation axes. The average relative movement between the tibia and calf shell in state PF25 in the x, y and z directions for the different displacements of the joint and rotation axes is shown in Figure 5.

For the relative movement between foot and foot shell (Figure 4), it should first be noted that the relative movement observed during the stance phase of gait is largely independent of axial displacement. The movements in the x and z directions are in the small millimeter range. In particular, the x-displacement (i.e., the displacement of the foot ‘forward' or ‘backward') shows a slight forward displacement (toward the toes) during the stance phase, which reverses into a backward displacement (toward the heel) toward the end of the stance phase. The z-displacement remains almost constant and shows virtually no change from the zero point. The most noticeable movement is in the y-direction. After a small peak at the beginning of the stance phase in the positive y-direction (in which the foot shell moves higher than the foot), a deflection in the negative y-direction occurs toward the end of the stance phase, which can be up to 20 mm. This deflection reduces again toward zero by the end of the stance phase.

During the swing phase of the gait cycle, differentiated results are evident between the various axis shifts. At the zero, inside and outside positions, there are virtually no significant deflections in any direction. At the up and down positions, only minimal differences are observed. For Up, a positive deflection in the y-direction of a few millimeters can be seen during the swing phase, while Down shows a negative deflection in the same direction. The most striking differences are seen in the Back and Front positions. For Back, the y-deflection increases positively to up to 10 mm during the swing phase, with the x-deflection also increasing negatively to up to 5 mm toward the end of the swing phase. Front, on the other hand, shows a negative y-deflection, which reaches its peak of 10 mm approximately in the middle of the swing phase. The x-deflection shows a negative trend until approximately the middle of the swing phase, which then reverses into a positive x-deflection until the end of the swing phase.

In the relative movement between the tibia and calf shell (Figure 5), it can be seen that the relative movement remains almost constant throughout the entire stance phase. Only toward the end of the stance phase do noticeable changes occur, showing a positive x-deflection (i.e., the calf shell moving closer to the tibia bone) of up to approximately 5 mm and a negative y-deflection (a downward shift of the calf shell) of up to approximately 2 mm. A notable deviation is the Up condition, in which a negative x-deflection of approximately 5 mm can be observed throughout the entire stance phase. In addition, Down shows a differentiated deflection, as the positive x-deflection begins much earlier in the stance phase, rises continuously and finally reaches a peak of approximately 8 mm. During the swing phase, a slight positive peak in the y-deflection of approximately 5 mm and a negative peak in the x-alignment of approximately 8 mm are also visible. However, there are hardly any noticeable differences between the Zero, Inside and Outside states. With regard to the Back and Front states, there is a variation in the amplitude of the respective peaks in the swing phase: while the deflections are reduced in Front, the peaks are more pronounced in Back. The most significant differences can be observed in the Up and Down states. In the Up state, the largest peaks occur for the positive y deflection (approx. 10 mm) and the negative x alignment (approx. 17 mm). In contrast, no peaks can be observed for Down. Rather, a continuous slight positive x deflection of approximately 1.5 mm and a slightly negative y deflection of approximately 3 mm can be observed throughout the entire swing phase.

**Figure 6:**
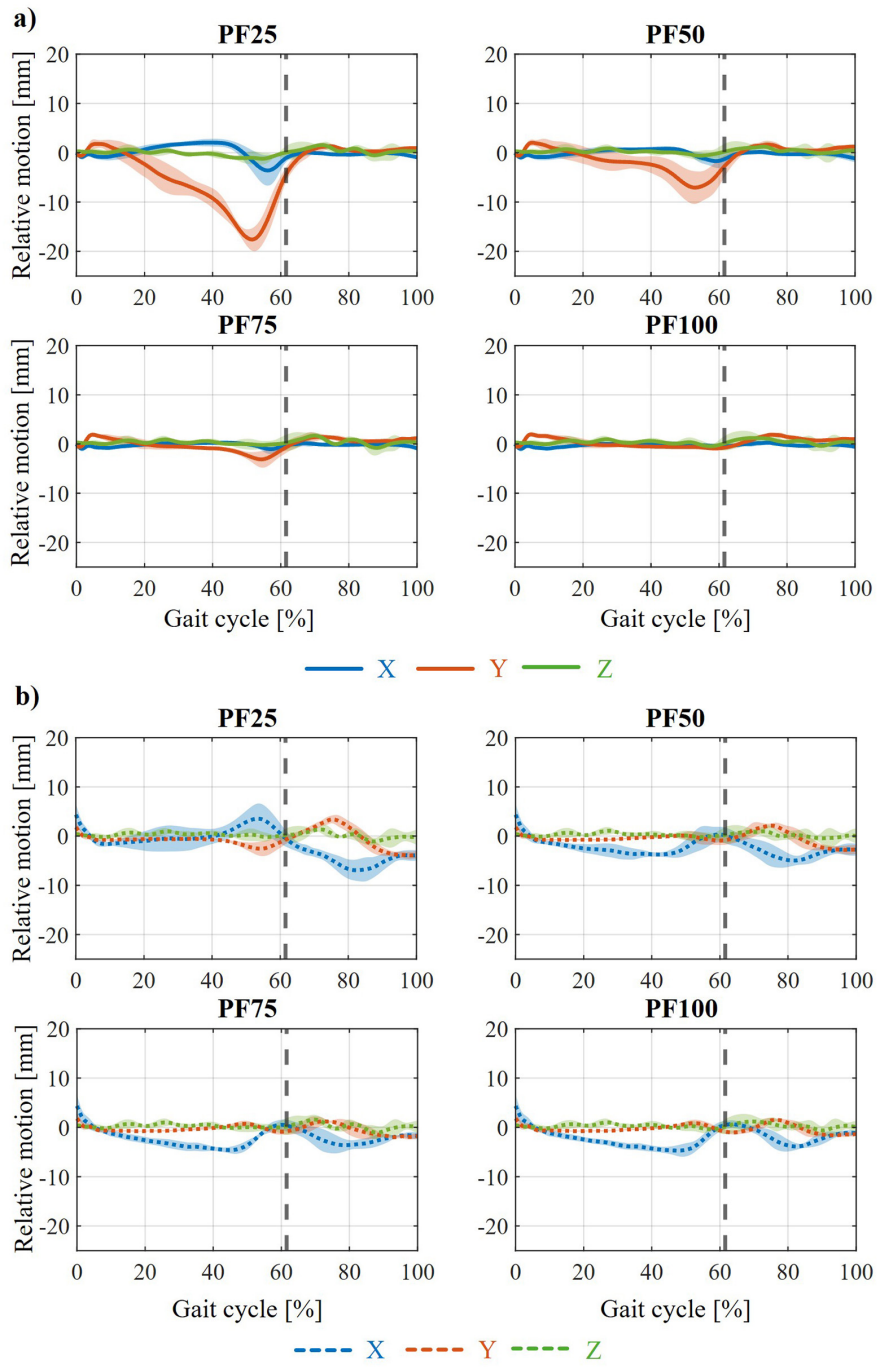
Resulting average relative movement for zero across the different weakening states and slow walking speed between **(a)** foot and foot shell and **(b)** tibia and calf shell.

**Figure 7:**
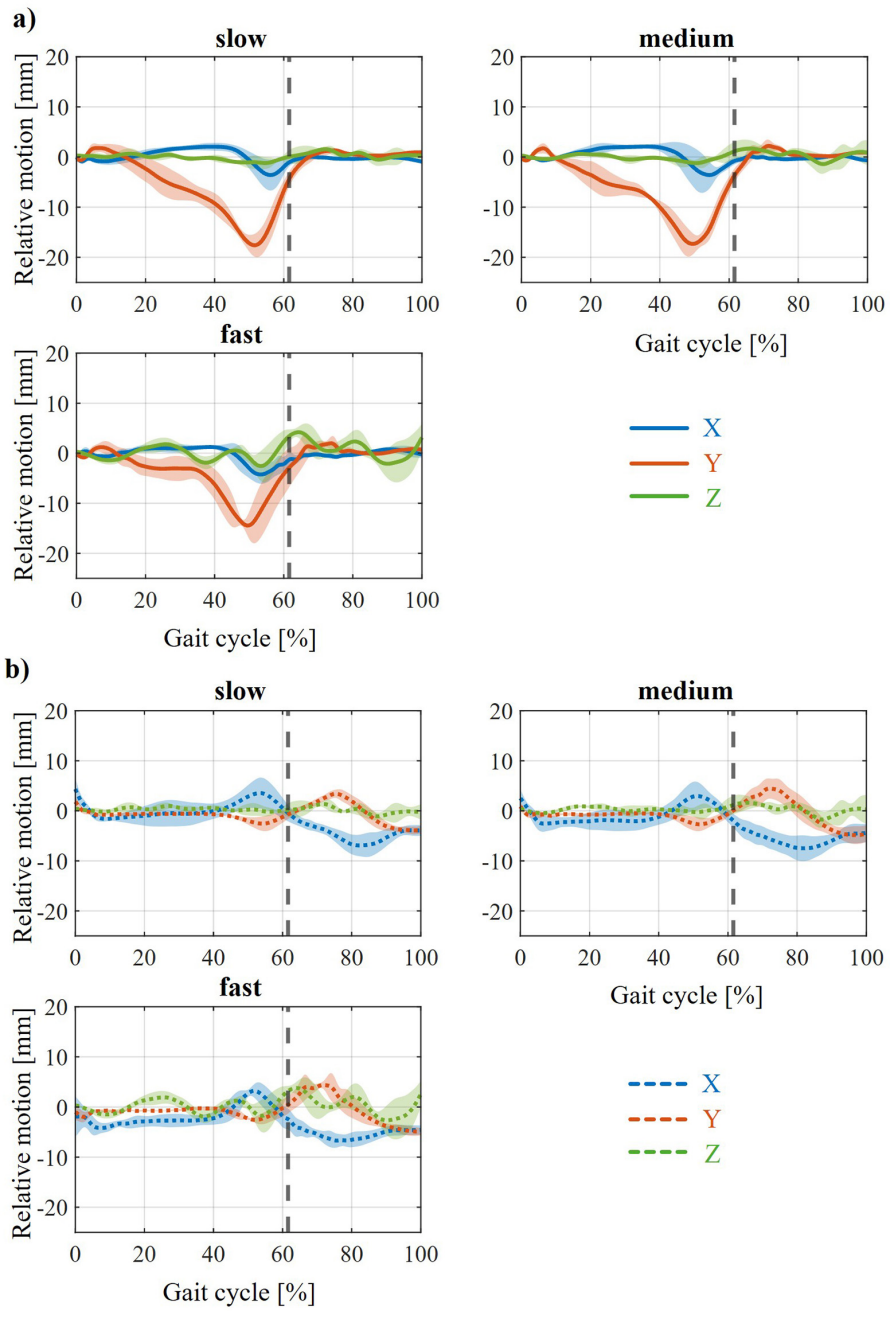
Resulting average relative movement for zero at PF25 at different walking speeds between **(a)** foot and foot shell and **(b)** tibia and calf shell.

The influence of various states of weakness (PF25–PF100) on the relative movement that occurs during zero displacement is shown in Figure 6. For the relative movement between the foot and the foot shell (Figure 6a), a reduction in the peak in the negative y-direction toward the end of the stance phase with increasing muscle strength (from PF25 to PF100) is most clearly visible. Furthermore, the amplitudes in the positive and negative x-direction also continue to decrease with increasing muscle strength. For the relative movement between the tibia and calf shell (Figure 6b), this attenuation is also evident from PF25 to PF50. In this case, a negative x-deflection even occurs predominantly in the stance phase. For the further weakening levels (PF75 and PF100), there are practically no differences to PF50. The different shifts between the ankle joint and orthosis axis show the same effects with varying degrees of weakening of the MHM. These are summarized in the Supplementary Figures S1–S6. The influence of the three walking speeds on the relative movement that occurs in the weakened state PF25 and the zero axis shift is shown in Figure 7. It can be seen that, with regard to relative movement, there is no discernible effect dependent on walking speed between the foot and foot shell (Figure 7a) or between the tibia and calf shell (Figure 7b). Except for increasing minor fluctuations in the z-direction with increasing walking speed, the curves are almost identical. An example of the same effect of different walking speeds can be seen in the Supplementary material for the front and up axis shifts (Supplementary Figures S7, S8).

### 3.2 Muscle activation

**Figure 8:**
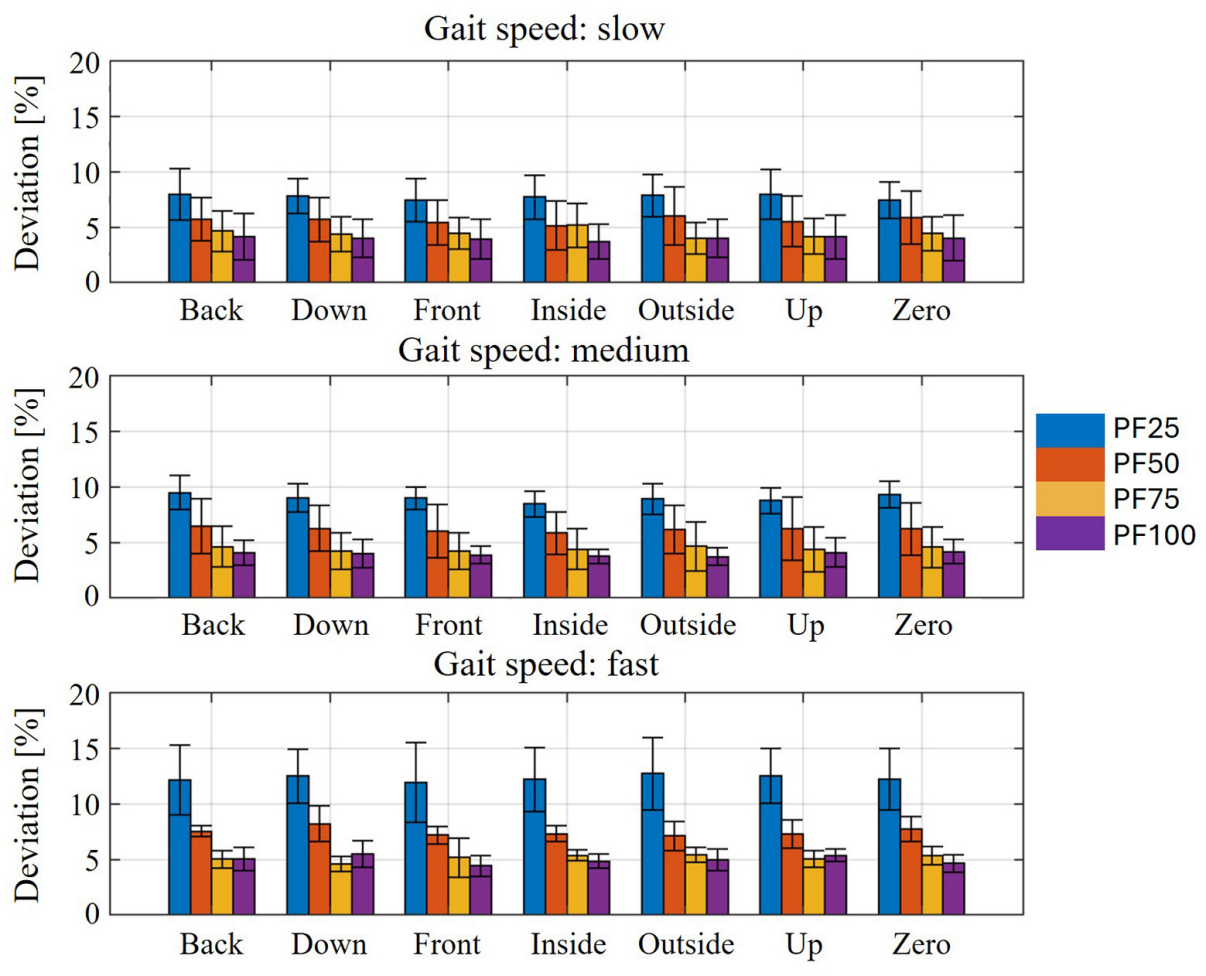
Average muscle activation deviation for different shifts of the orthosis and joint axis for different degrees of weakness in all test subjects compared to the optimal plantar flexor activation curve in the stance phase at the three gait speeds.

The resulting relative movement between the orthosis (or the individual shells) and the MHM causes a change in the point of application of the support provided by the orthosis. As a result, the muscle activation of the individual models is influenced by the force support. The effect of the yielding behavior of the biological soft tissue on the activation of the plantar flexors in the individual states of weakness is shown in Figure 8. This shows the average deviation from the muscle activation curve determined using the assistance-as-needed method (Scherb et al., 2023b) in the standing phase across all test participants. The average deviation in plantar flexor muscle activation shows that, for all different states of axial displacement, the deviation from the target state increases as a result of biological soft tissue modeling with decreasing residual muscle strength. Thus, the slow walking speed at PF25 shows the largest mean deviation of up to approx. 8%. At PF100, the lowest mean deviation of all weakening conditions can be seen at just under 4%. Furthermore, the shift in the ankle joint and orthosis axis has no clearly discernible effect on the average deviation from the target state. Minor exceptions can be seen in the Inside shift, where the average deviation is approximately the same for PF50 and PF75. Another exception can be seen in the outside and up shifts, where the average deviation is the same for PF50 and PF25. However, these effects are no longer observable when considering the higher walking speeds (medium and high). Overall, the same effect can be seen at higher walking speeds as at slow walking speeds, namely that the average deviation increases with decreasing muscle strength. Here too, when walking speeds are considered in isolation, no real influence of the axis shifts on the average deviation can be seen. The average deviation is roughly the same for all axis shifts for each state of weakness. A comparison of the different walking speeds shows an increase in the average deviation with increasing walking speed. In the case of PF25, this increases from 8% to 10% (average walking speed) and up to 13% (fast walking speed). A similar behavior can be observed for the other attenuation levels.

**Figure 9:**
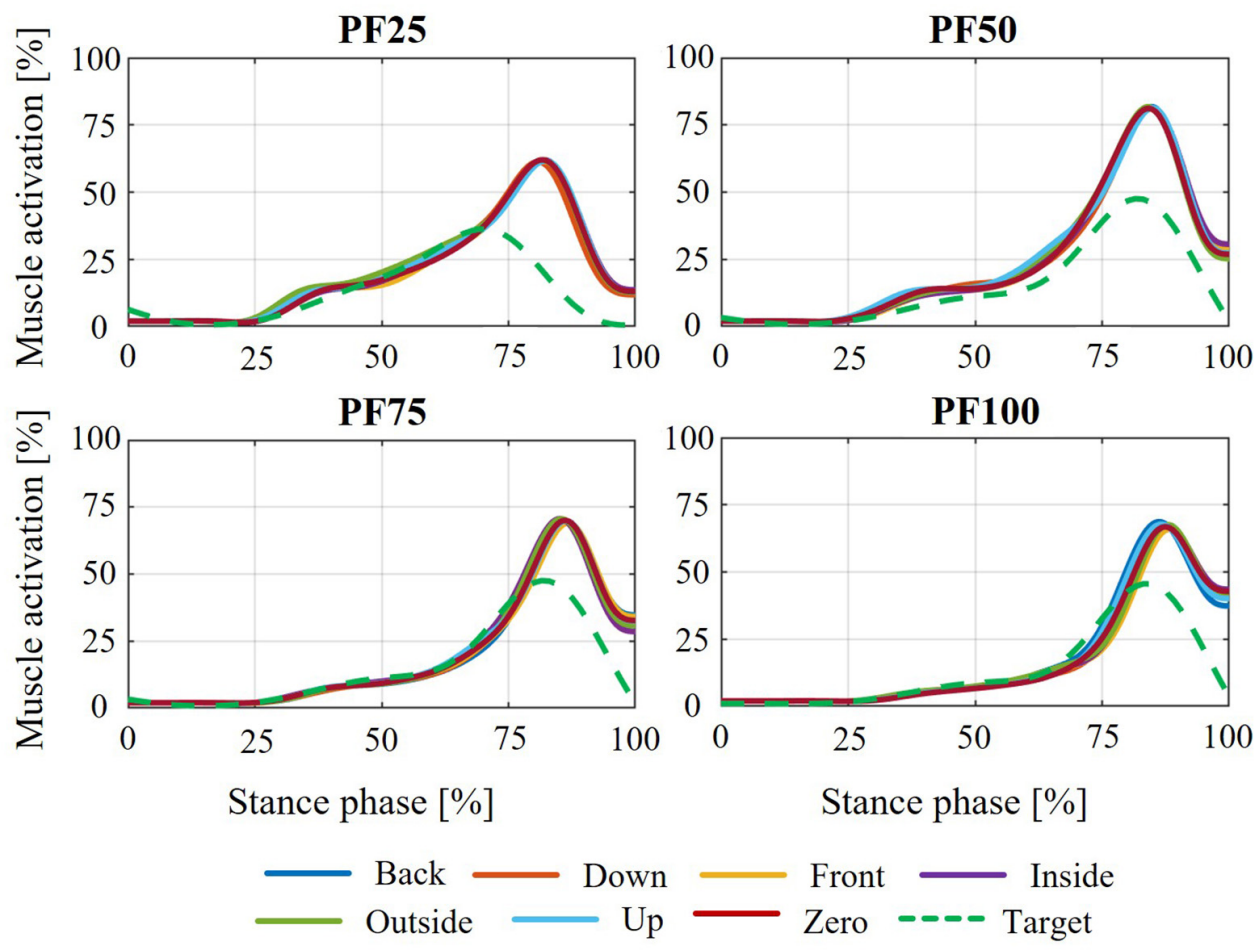
Resulting muscle activation curves of the plantar flexors depending on the various axial shifts for P12 in the five weakening states (PF25 to PF100) and slow walking speed during the stance phase of the gait cycle.

**Figure 10:**
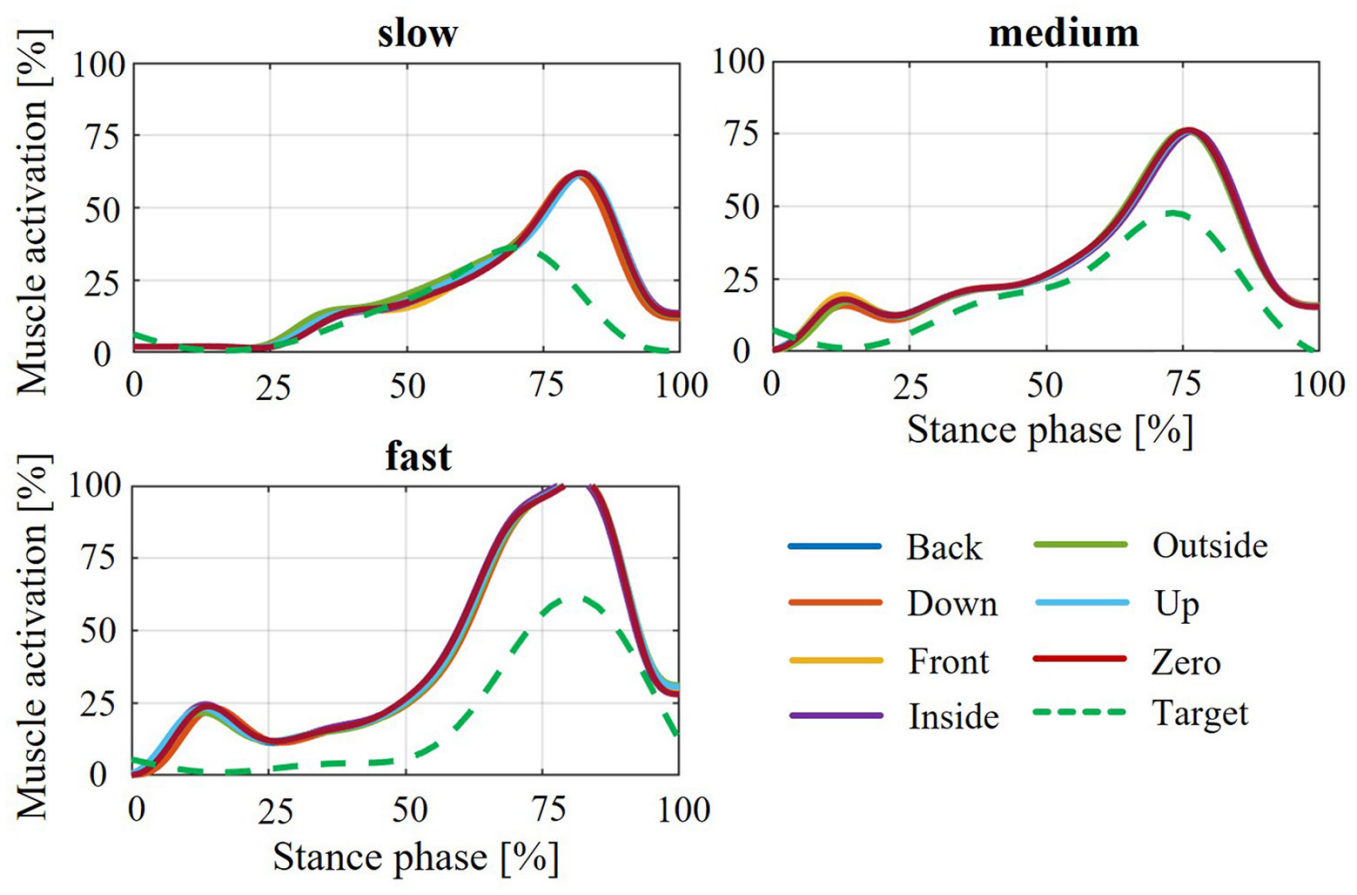
Comparison of the resulting muscle activation curves of the plantar flexors depending on the axial shifts at P12 with PF25 between the three walking speeds over the stance phase of the gait cycle.

The findings for the average deviation in muscle activation are also evident when observing the course of plantar flexor muscle activation during the stance phase of the gait cycle. To show this, the exemplary activation curves of the captured participant no. 12 (P12) are used. There is an increasing deviation in the muscle activation curve of the plantar flexors from the targeted curve from PF100 to PF25 (Figure 9). The different shifts of the orthosis to the ankle joint axis do not result in any noticeable differences in the activation curve. A comparison of the plantar flexor activation curve from P12 to PF25 at different gait speeds also shows an increasing deviation from the target activation curve with increasing gait speed (see Figure 10).

## 4 Discussion

### 4.1 Relative movement

The simulation results of the biological soft tissue behavior in the human simulation with regard to the resulting relative movement show many similarities in the various axial displacements between the ankle joint and orthosis axis. Firstly, all variations and both analyzed positions show that there is hardly any deflection in the z-direction. This is also plausible, as the ankle joint and AFO axes were used as ideal axes running in the z-direction in the simulation performed. This is due to the initial definition in the MHM used. Accordingly, there is also practically no effect from a shift of the AFO axis to the ankle joint axis in the z-direction (inside and outside) compared to the initial state (zero). Furthermore, a slight positive y-deflection between the foot and the foot shell (Figure 4) can be seen in each variation. This indicates the penetration of the AFO into the sole of the foot in order to counteract the sinking of the foot after the HS and thus achieve a controlled lowering of the foot at the beginning of the gait cycle. Furthermore, for each variation, the maximum y-deflection in the negative direction is evident toward the end of the stance phase. This represents the penetration of the orthosis into the foot (more precisely, the instep) in order to enable forward acceleration when walking through the moment applied by the AFO.

Furthermore, the relative movement between the tibia and calf shell (Figure 5) shows a positive x-deflection toward the end of the stance phase in every variation. This can also be seen at the point of maximum force application by the orthosis during the gait cycle and means that the calf shell penetrates the tibia (or, in reality, more accurately, that the Velcro fastener penetrates the tibia) in order to provide the necessary support. Together with the movement of the foot shell, this results in the AFO unfolding to provide plantar flexion support. Furthermore, a negative x-deflection can be seen in the swing phase, which means that the calf shell penetrates the calf to support the dorsiflexion of the ankle joint during the swing phase. The differences in the relative movement between the foot and the foot shell can be seen at the back and front (Figure 4). The negative (back) and positive (front) x-displacement at the end of the swing phase is probably due to the defined initial axis displacement. The change in y-deflection in these two variations, on the other hand, is due to the change in kinematics between the AFO and the foot or leg and the corresponding change in the lever arm in the plane of moment application. The main differences in the relative movement between the calf shell and tibia can be seen in Up and Down (Figure 5). The explanations for the changes in x and y deflection in these variations are analogous to the previous explanations between the foot and the foot shell. The different peaks when comparing the various weakening states (Figure 6) are also considered plausible. The varying degrees of support applied in the different states result in different degrees of penetration of the AFO into the biological soft tissue. Thus, in the weaker models (PF25), the AFO exerts greater force on the person, compressing the biological soft tissue more strongly and thus creating greater relative movement between the AFO and the leg or foot. This is particularly evident in the stance phase in the various states of weakness, as this is where the different strengths of the plantar flexors have the greatest effect as a difference between the MHMs. Ultimately, the orthosis guides the patient rather than being passively carried along, which is also in line with the actual purpose of the orthosis. The minimal influence of walking speeds on relative movement is also an expected effect (Figure 7).

The plausibility of the simulated results is further supported by comparison with previously published findings. A maximum deviation of approximately 20 mm between the WAD and the human limb was also reported by Lee et al. (2014), indicating consistency with experimental observations. These peak deviations occur at specific phases of the gait cycle, namely during stance and swing, when the WAD provides maximal assistance for plantarflexion and dorsiflexion, respectively (Alvarez et al., 2017; Langlois et al., 2018). Moreover, the dependence of the relative motion between the human limb and the WAD on the level of assistance—arising from differences in joint kinematics between the biological joint and the device joint—has been documented in prior studies (Torricelli et al., 2018; Ballen-Moreno et al., 2022). Taken together, these consistencies with the existing literature substantiate the principal validity of the simulation results and confirm that the main observed effects align with experimentally established findings.

### 4.2 Muscle activation

The effects caused by modeling the yielding biological soft tissue behavior in relation to relative movement also have an impact on the resulting muscle activation of the plantar flexors. It should be emphasized again at this point that the comparison was made with the muscle activation curves from the support determination method. The intention here is not to classify one of the two models as more correct than the other, but rather to illustrate the deviations in the activation curves of the plantar flexors. These deviations therefore require different conclusions to be drawn with regard to the design and configuration of the AFO in practical application. Consequently, the resulting relative movement and the resulting change in the point of application of the force transmission result in deviations in muscle activation (see Figures 8, 9). The strongest muscle activation deviations occurring in PF25 are due to the largest relative movements between the orthosis and the leg or foot (Figure 6). Furthermore, no real effects on muscle activation deviation can be detected for the individual shifts between the ankle joint and orthosis axis, as these are relatively similar and the differences for individual shifts have no significant effect on the resulting muscle activation. The increasing deviation in muscle activation in the comparison curve with increasing gait speed (Figures 8, 10) despite a similar relative movement can be explained by the stronger torque in the ankle joint over the gait cycle with increasing gait speed.

In addition, the occurring residual forces were investigated in the simulations. These are virtual forces typically introduced into the simulation to ensure dynamic consistency (Wechsler et al., 2024). However, OpenSim provides boundary values to help evaluate the correctness and plausibility of the executed simulations (Hicks et al., 2015). The residual forces and moments for the soft tissue simulations are higher for all test subjects, walking speeds and axial displacements compared to the simulations used to determine the support curve (Scherb et al., 2023b). They even approach the specified limit values by OpenSim, but do not exceed them. The increases can probably be explained by the dynamics of the AFO within the human simulation as the captured motion was from healthy humans without any assistive system. It is further not possible to define a pattern in the influence of the various axis shifts on the activation of the residual actuators. Thus, these effects for the residual forces underline that the simulation results of the biological soft tissue simulations show a plausible behavior.

### 4.3 Limitations

The first limitation is that simulation of biological soft tissue behavior is a pure simulative approach. In order to clarify that the simulated behavior that is closer to reality, future research should aim to validate the simulated relative movement between WAD and MHM. Despite the plausible results of the relative movement that has been generated, these should be compared with the resulting movements in real humans and WAD prototypes. This could be achieved using motion capture recordings, in which the movements of the human and the WAD can be determined individually using applied markers (Childers and Siebert, 2016). However, this requires a certain amount of effort in separating the individual movements and a certain degree of high quality in recording accuracy. An evaluation using IMU systems would also be conceivable, for example by attaching a sensor to the WAD. However, due to the underlying inaccuracy in the recording of movements (Abhayasinghe et al., 2019) compared to optical recording systems, further development of the technical standard is still required. Furthermore, the relative movement between the WAD and the human body could be recorded by attaching a laser motion sensor to the WAD. This has already been used for a gait orthosis to record the 2D movement of the orthosis (Noll et al., 2018).

**Figure 11:**
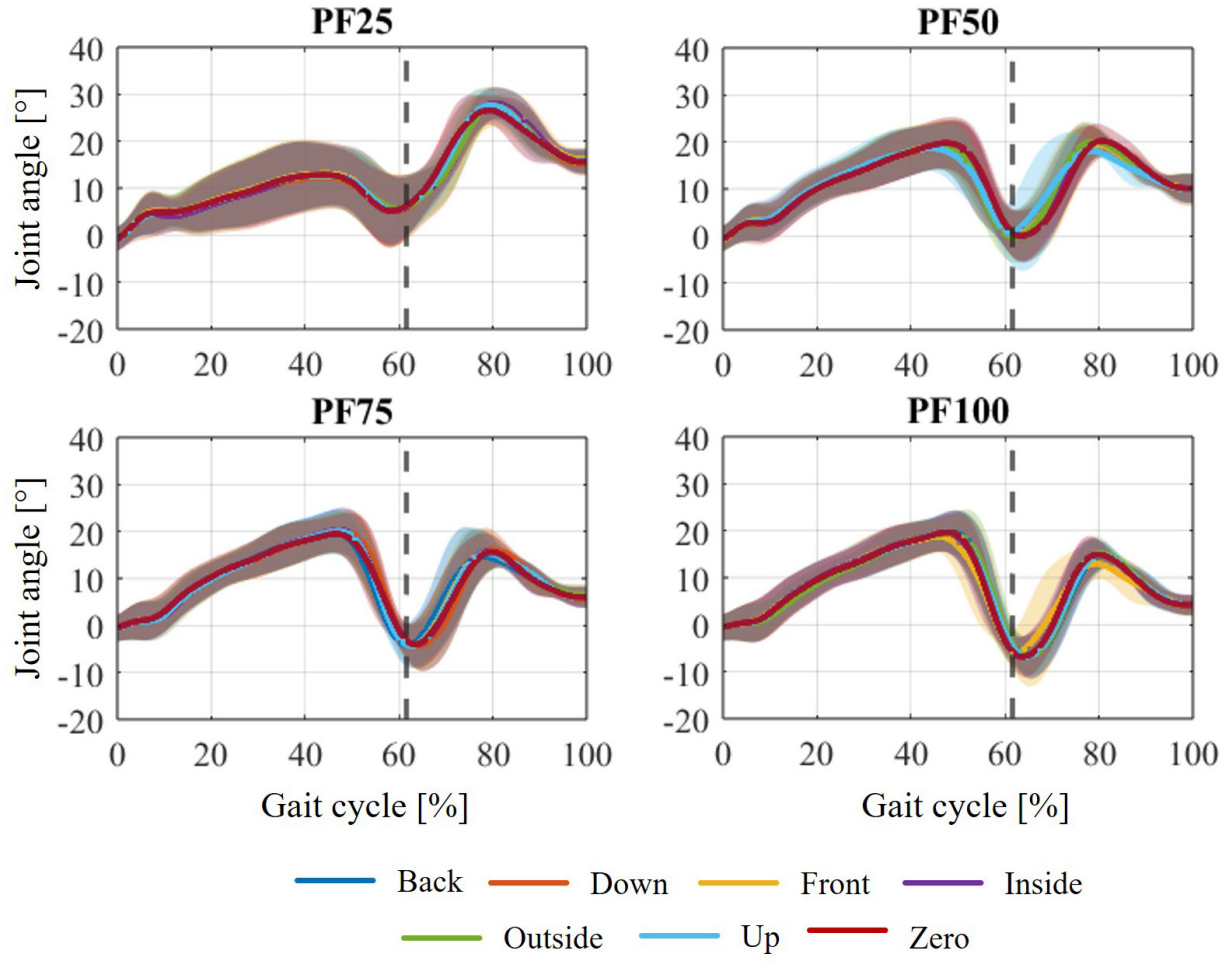
Comparison of the resulting ankle joint angle due to CMC for all weakening levels in the variations of the axis displacements.

The simulation of PF100 (Figure 9) shows an anomaly in the form of a noticeable deviation from the reference curve despite there being hardly any relative movement. To explain this effect, the resulting ankle joint angle after the CMC must be considered. Since the CMC in the OpenSim simulation pool represents the first step toward a forward dynamic simulation, the specified joint angles are optimized or changed within it as needed. In the case of missing required joint angles over the gait cycle, as for the two shells of the AFO, this property is suitable for determining the movement behavior. However, one disadvantage is that the specified joint angles can also be adjusted considering the boundary conditions. In this case, the joint angles in the leg, mainly of the ankle joint, are adjusted by applying the orthotic support within the CMC. This effect can be mitigated by increasing the weighting for maintaining joint angles. In this simulation, the weighting of the ankle and knee angles was increased to 10 (compared to 1 for the other angles—meaning that these entered angles should be retained with 10 times greater importance) to minimize the effect of the angle change and the corresponding change in gait pattern. When comparing the resulting ankle angles of the various axis shifts between the weakening states (see Figure 11), it can initially be seen that the individual axis shifts have no influence on the ankle angle change, but that this only depends on the weakening state and the applied support moment.

**Figure 12:**
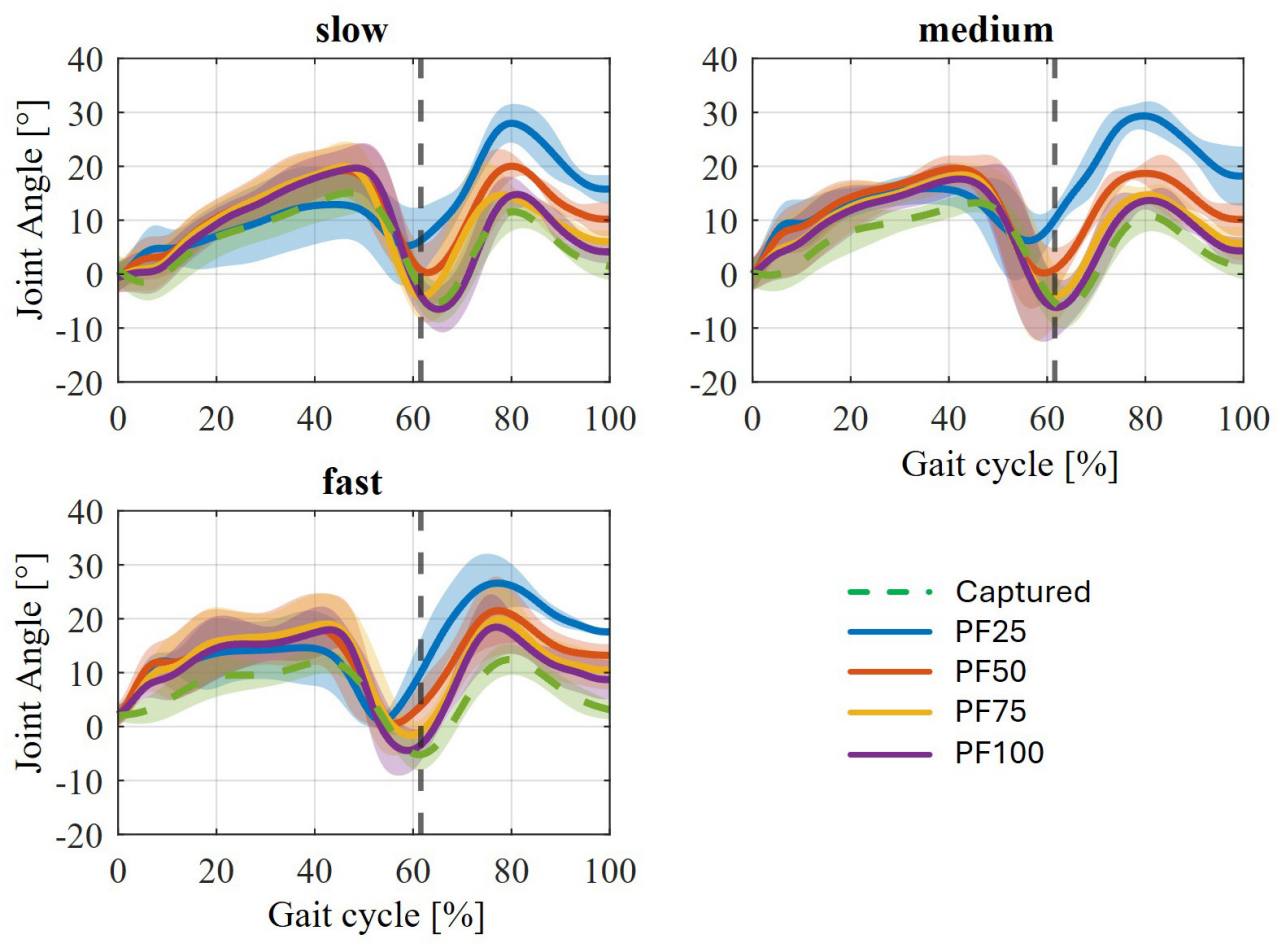
Comparison of the average resulting ankle angles due to CMC at different levels of weakness and the three walking speeds.

If now the resulting ankle angles for all variations in axial displacement at the different levels of weakening with the joint angle recorded from the motion capture data are compared (Figure 12), clear deviations can be observed. For PF50, PF75 and PF100, the most deviations occur in the stance phase, whereas for PF25, the deviations are most pronounced in the swing phase. The same effects can be seen across the different walking speeds. The change in the ankle joint angle in the swing phase for PF25 once again underlines the pure focus of the previous analysis on the stance phase. Consequently, the curves showing the muscle activation of the plantar flexors (Figures 9, 10) always show an increase in activation at the end of the stance phase. This describes a necessary activation of the plantar flexors in the swing phase, which is required to regulate this deflection due to the high dorsiflexion angle. Thus, the resulting changes in muscle activation of the plantar flexors during the stance phase can be explained by the relative movements that occur, the change in joint angle, and the combination of both.

The change in joint angles that occurs represents a certain limitation in that it makes comparison with previous examinations difficult. In reality, the change in joint angles and overall movement behavior during gait is the intended purpose of AFO support. The treatment of patients with foot drop aims to influence their pathological gait pattern as a result of orthotic treatment toward healthy gait behavior. In this case, the problem lies in the underlying use of gait data recorded from healthy participants and the assumption that the change in movement behavior is already implicitly included in the AFO. However, this is not taken into account by the CMC. An improvement in the movement achieved from the CMC could be achieved by adjusting the set tasks for tracking the joint angles. Alternatively, patient movement data would have to be used or a forward dynamic simulation in the sense of optimal control [Dorschky et al., 2019; e.g., trajectory optimization (Winkler et al., 2018)] would have to be applied.

Another limitation is the direction-independent adjustability of stiffness and damping within the BushingForces (Table 1). This means that different stiffness values would have to be applied for negative and positive deflections in the individual spatial directions (x, y, and z) in order to simulate real behavior. This can best be applied in the x-direction of the tibia. A deflection in the positive x-direction means that the calf shell presses on the calf, while a deflection in the negative x-direction means that the calf shell or Velcro fastener presses on the shin. The same stiffness value is used for both deflections. However, the calf has a relatively large amount of biological soft tissue with a lot of muscle volume, which can be compressed more strongly. In contrast, there is only a thin layer of biological soft tissue in front of the tibia bone on the front of the tibia. Accordingly, a significantly higher stiffness would have to be used within the BushingForces in the negative x-direction than in the positive x-direction. Furthermore, the same set of bushing forces, including stiffness and damping values, is used for each simulation and for each test participant. However, the stiffness values depend on the individual biological soft tissue components (skin, fat, muscles) and their composition. Since this composition varies for each test participant and for each part of the body, the bushing forces would have to be adjusted individually each time. In addition to individual factors (e.g., weight), genetic factors and factors such as age, gender, or even ethnicity also influence the parameters to be selected (Borkan et al., 1983; Wang et al., 2000; Rush et al., 2009; Bosy-Westphal et al., 2011). In this context, there are various studies that analyze the composition of biological soft tissue at different parts of the body depending on certain influencing factors (Jebb, 1997; Levine et al., 2000). These could be used to generate a database that would allow individual adjustment of the bushing forces depending on the parameters of the test person. The aim of this study was to find an exemplary set of parameters that, in a first step, fulfills the intended functionality of relative movement estimation and does not require the creation of patient-specific simulations. Accordingly, this study can be used as a basis for further in-depth investigations in the field of biological soft tissue modeling.

### 4.4 Conclusion

Overall, it can be seen that the intended effect of simulating the yielding behavior of biological tissue is supported by the simulation results. By modeling the biological soft tissue using spring-damper elements at the interface between the MHM and AFO, relative movement can be generated. This is not limited to individual degrees of freedom, but allows for free movement in space. In addition, the resulting relative movements appear to be plausible overall. Furthermore, effects of the resulting shift in the point of application of the support provided by the AFO on the resulting muscle activation of the plantar flexors are apparent. This leads to the conclusion that the approach shown offers a suitable option for modeling the interaction between MHM and WAD. Accordingly, a further effect from reality can be taken into account in the interaction modeling of human simulation.

## Data Availability

The original contributions presented in the study are included in the article/Supplementary material, further inquiries can be directed to the corresponding author.
