# Supplementary material for: Integrating the behavior of biological soft tissue into musculoskeletal simulation for the design of wearable assistive devices
Source: Front Hum Neurosci. 2026 Mar 27;20:1781987. doi: 10.3389/fnhum.2026.1781987 (PMC13066241; doi:10.3389/fnhum.2026.1781987)
Supplement: Supplementary file 1 [file Data_Sheet_1.docx]

Supplementary Material

# Supplementary Data


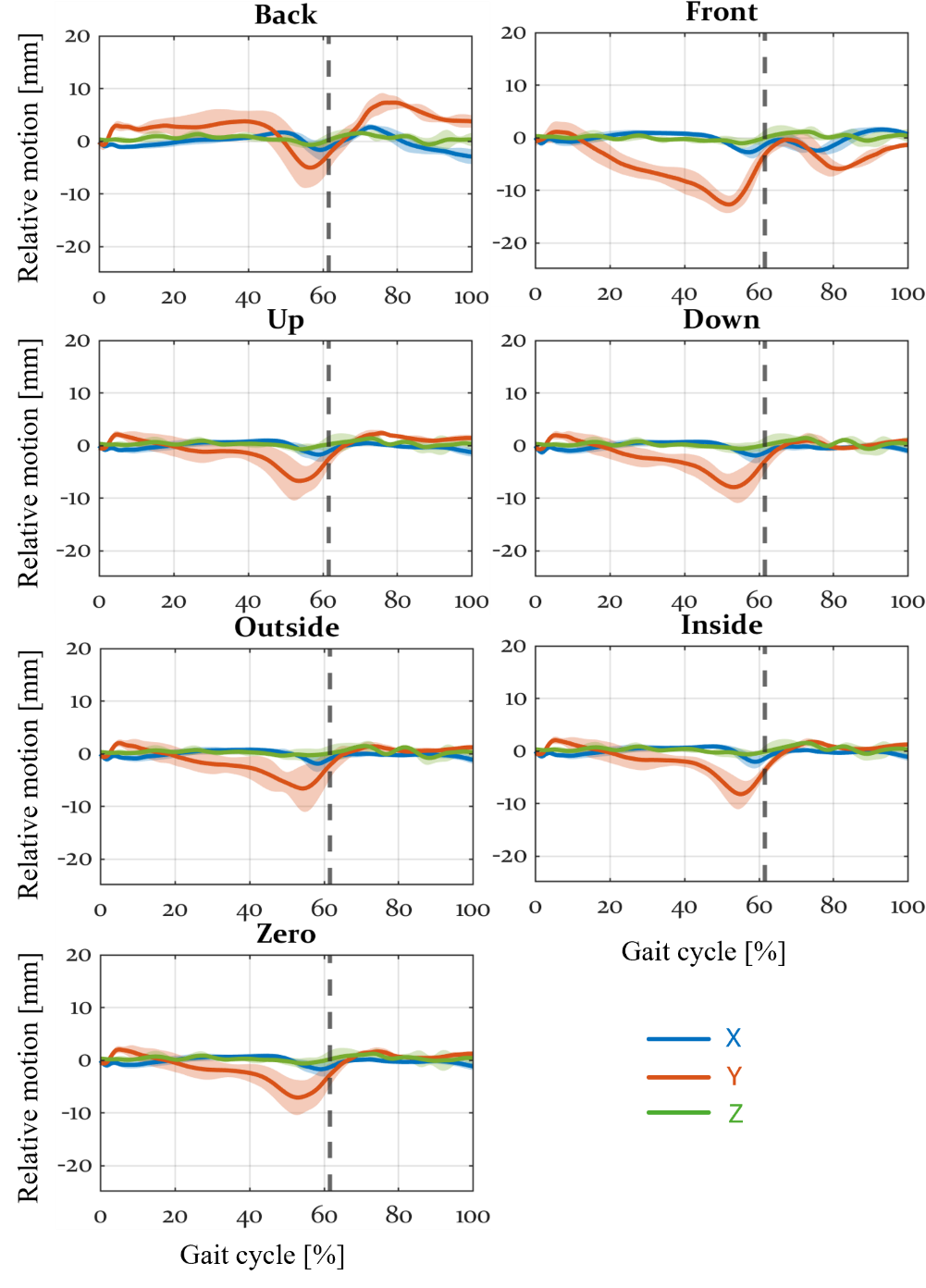


Figure S1: Resulting relative movement between foot and foot shell across all subjects with PF50 for the various shifts between joint and orthosis axis


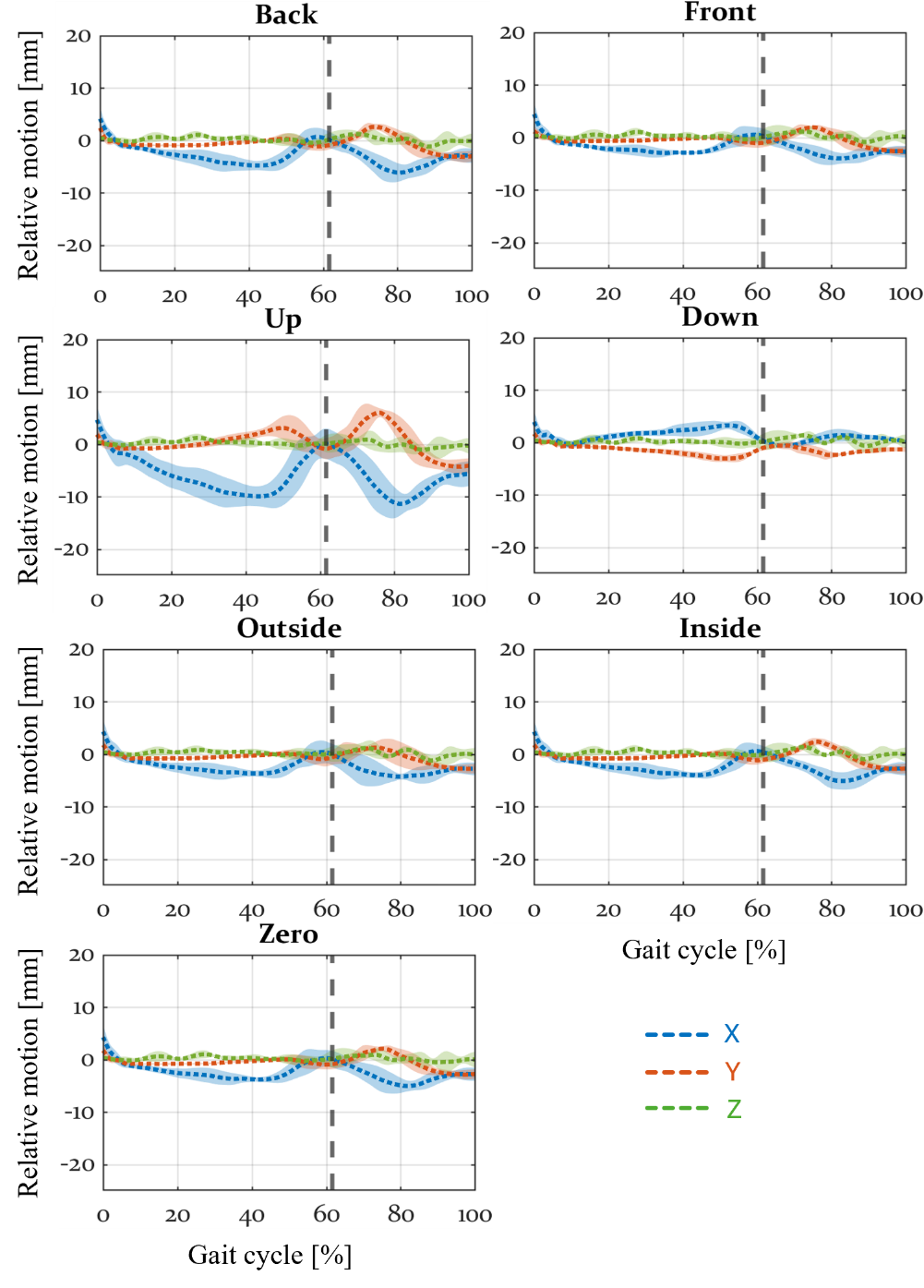


Figure S2: Resulting relative movement between calf and calf shell across all test subjects with PF50 for the various shifts between joint and orthosis axis


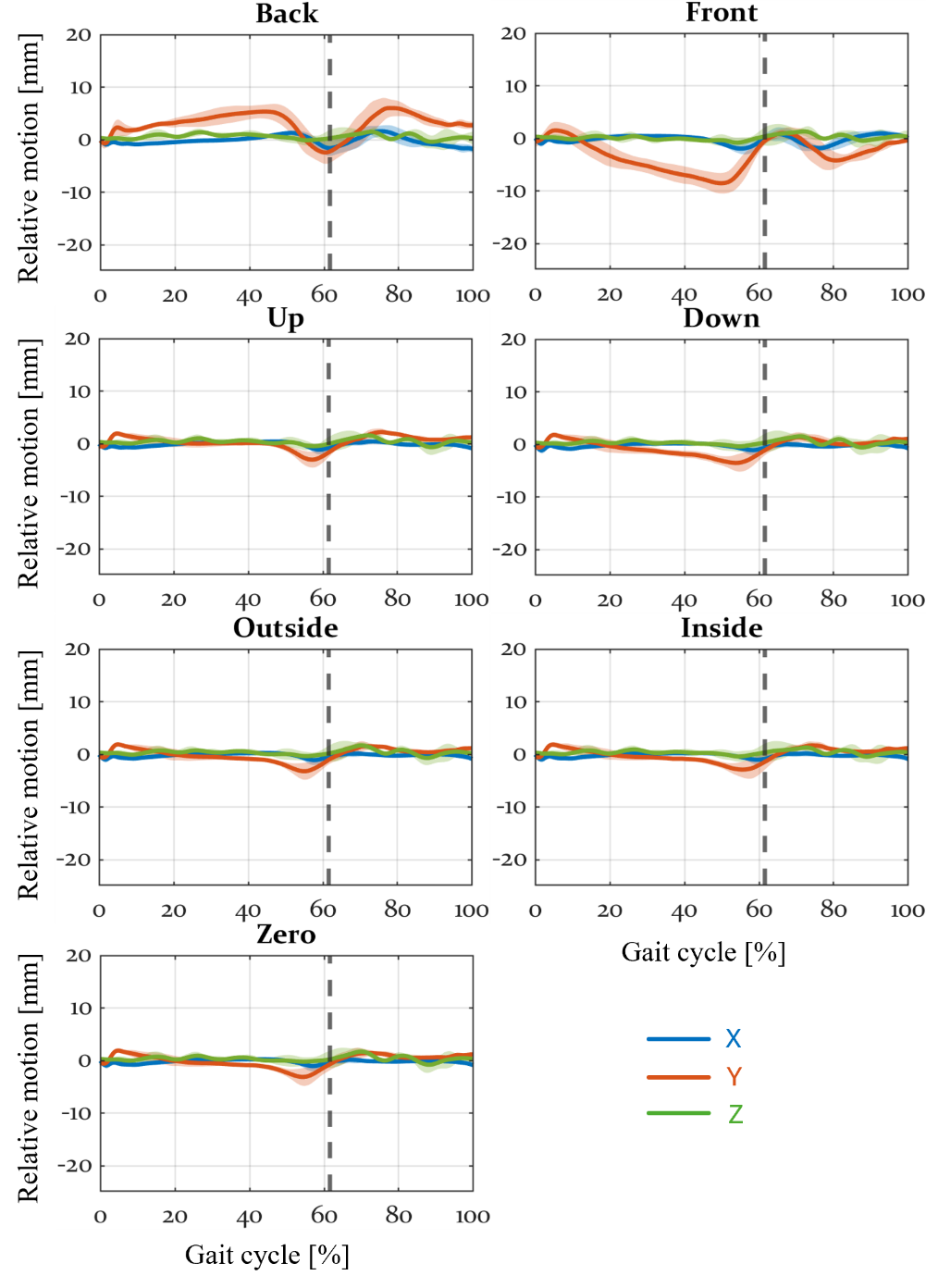


Figure S3: Resulting relative movement between foot and foot shell across all test subjects with PF75 for the various shifts between joint and orthosis axis


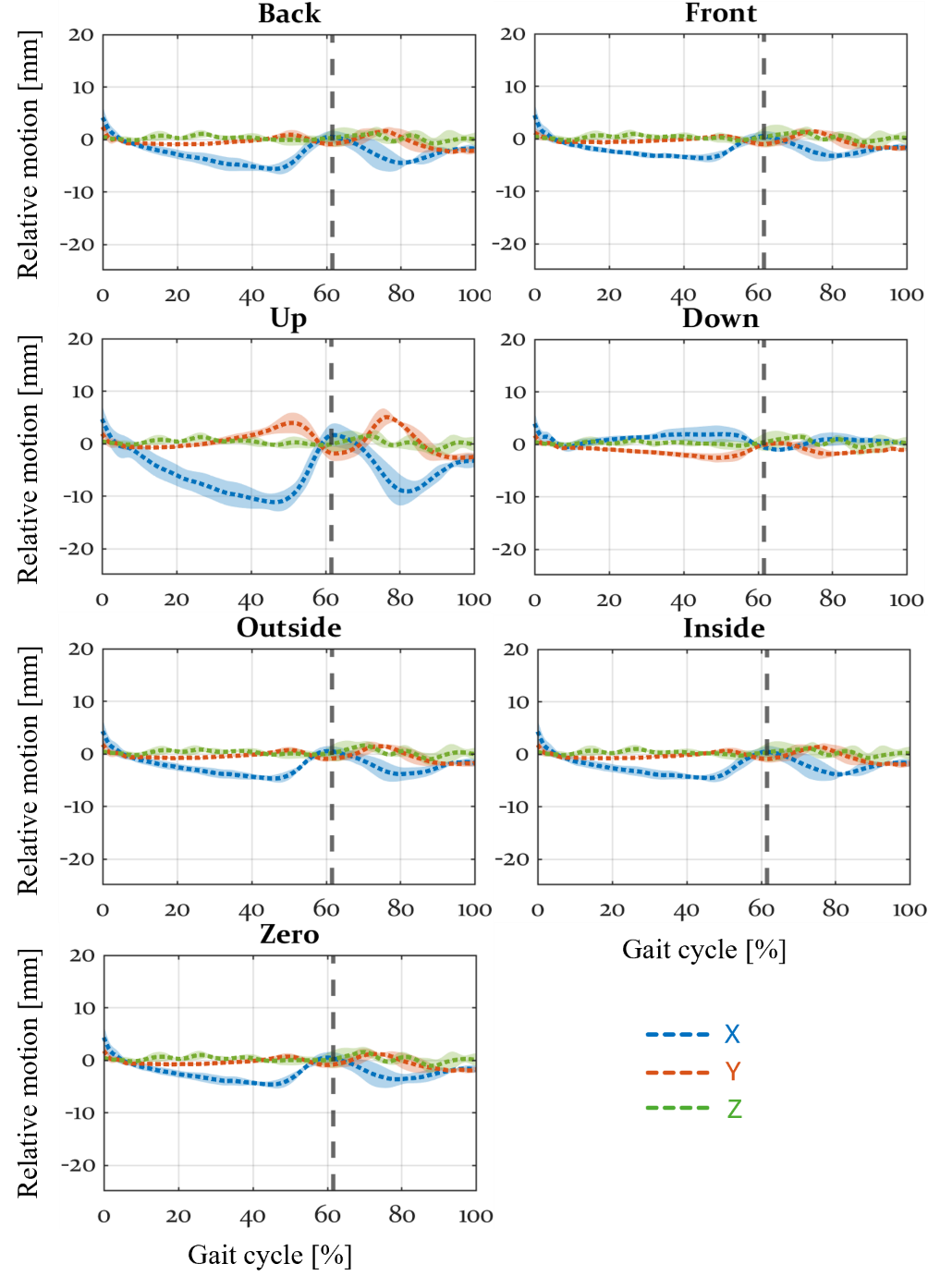


Figure S4: Resulting relative movement between calf and calf shell across all test subjects with PF75 for the various shifts between joint and orthosis axis


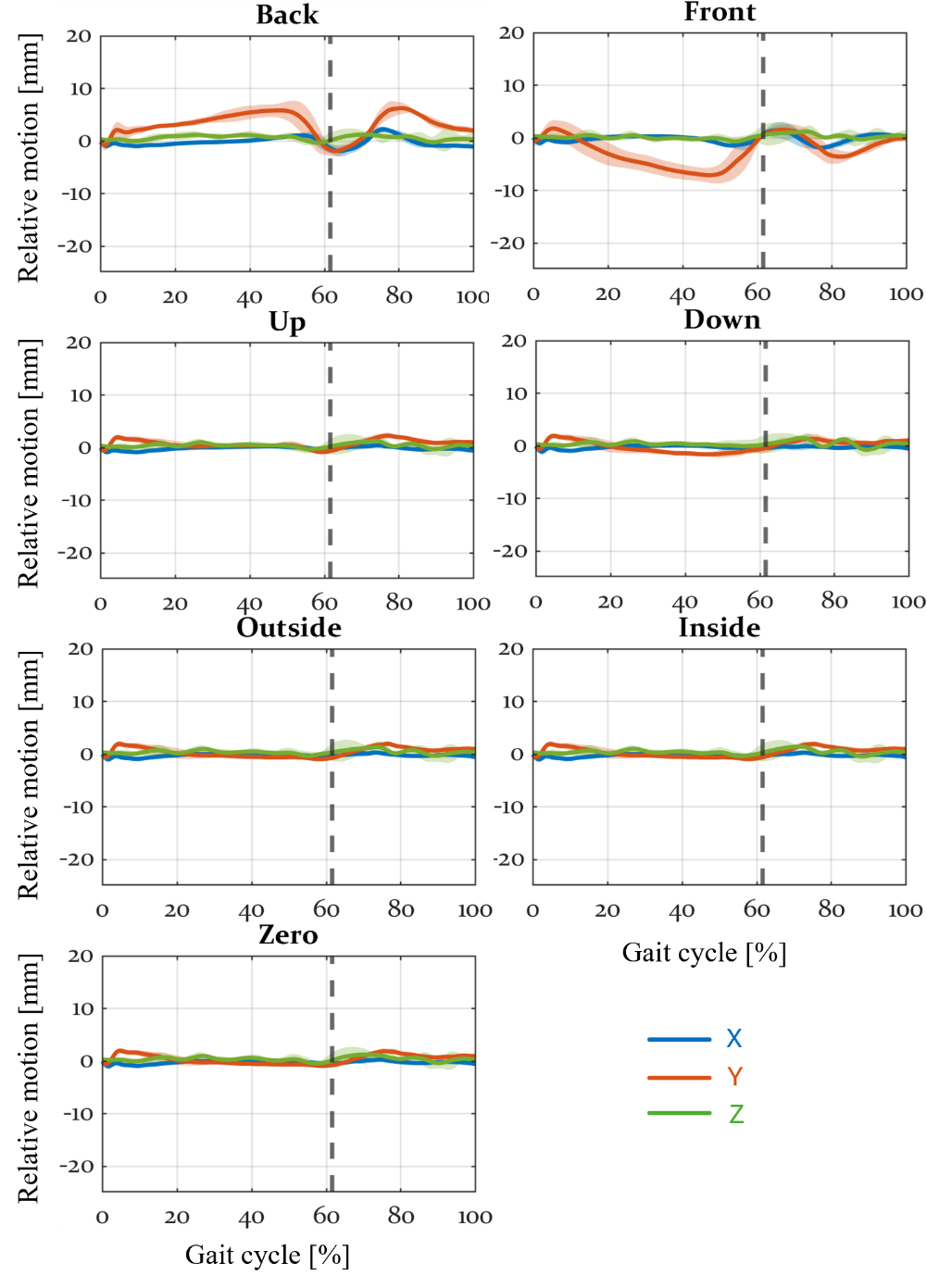


Figure S5: Resulting relative movement between foot and foot shell across all test subjects with PF100 for the various shifts between joint and orthosis axis


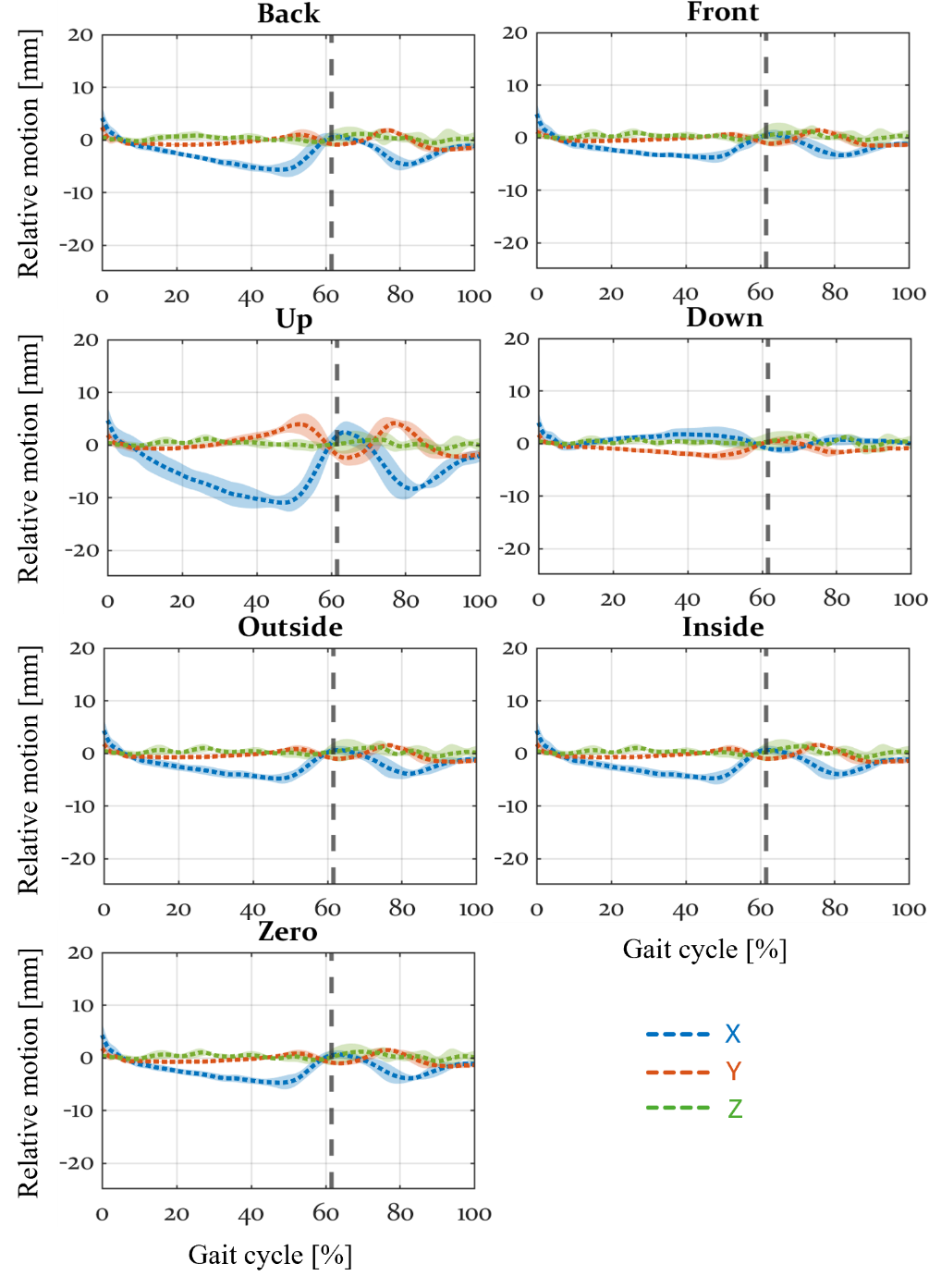


Figure S6: Resulting relative movement between calf and calf shell across all test subjects with PF100 for the various shifts between joint and orthosis axis


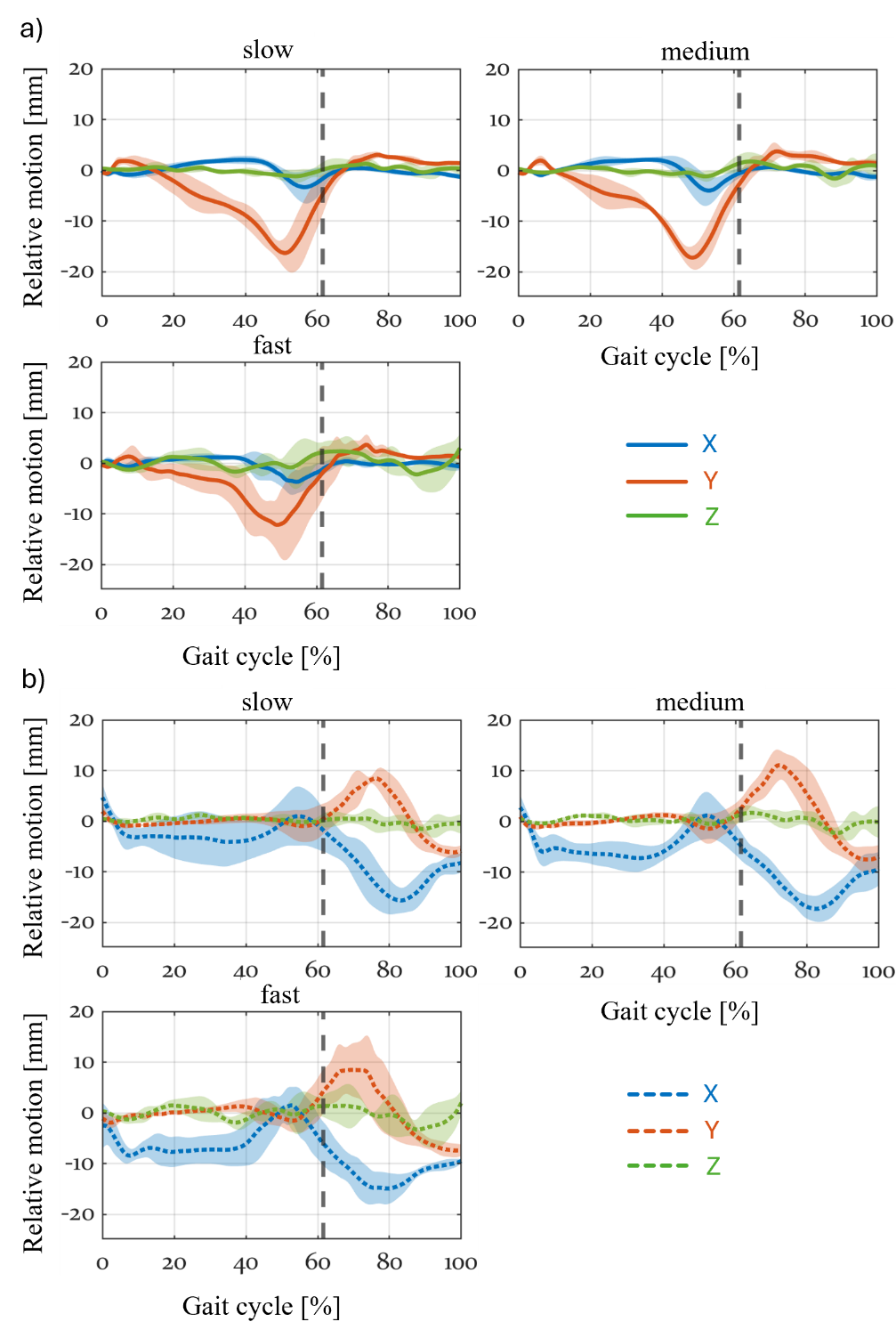


Figure S7: Resulting average relative movement for Up at PF25 at different walking speeds between a) foot and foot shell and b) calf and calf shell


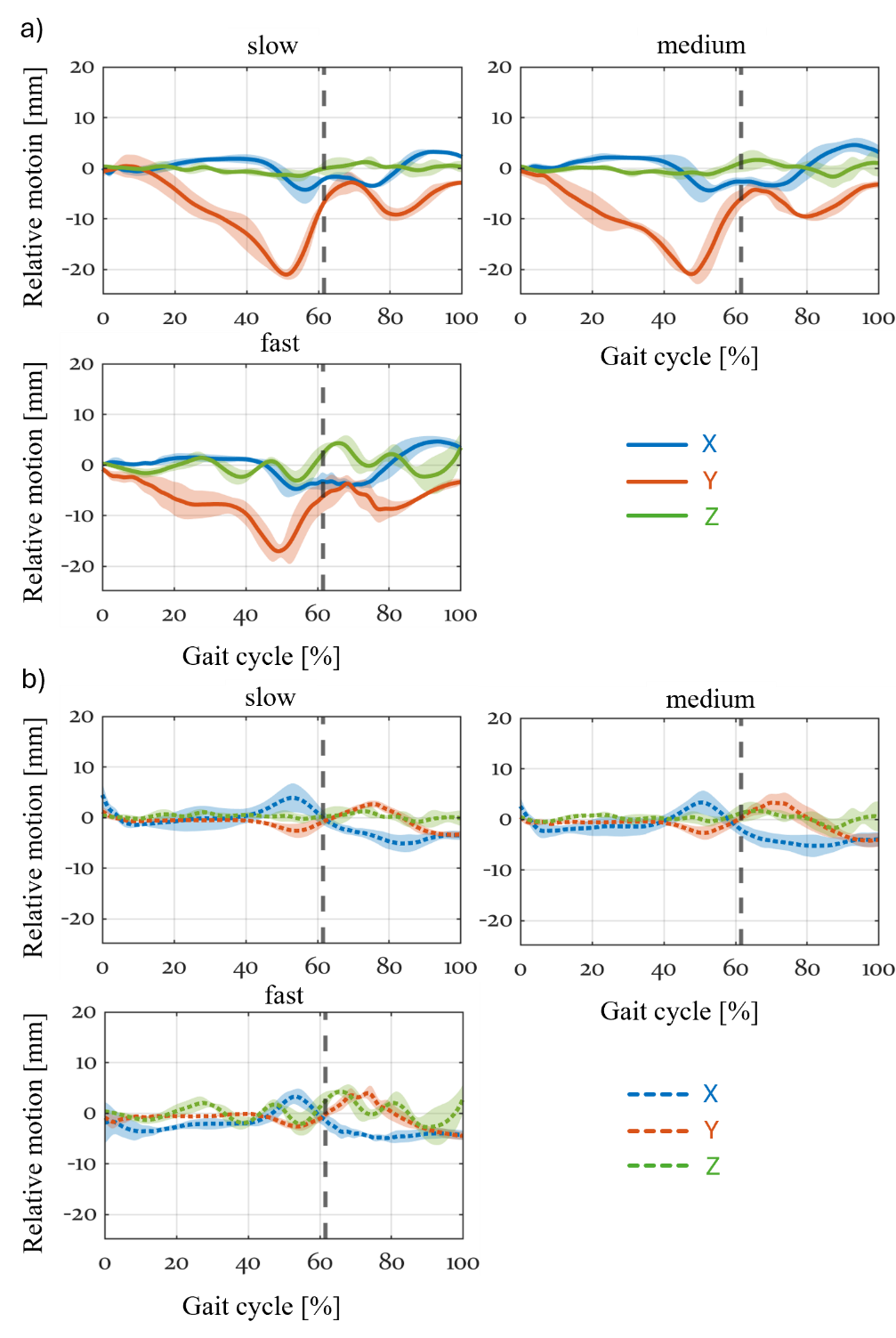


Figure S8: Resulting average relative movement Front at PF25 at different walking speeds between a) foot and foot shell and b) calf and calf shell
